# A *Ralstonia solanacearum* effector regulates plant cell death by disrupting the homeostasis of the BPA1-ACD11 complex

**DOI:** 10.1128/mbio.03665-24

**Published:** 2025-02-25

**Authors:** Bingbing Xue, Yan Zhou, Yongxiao Xie, Xiaocheng Huang, Jinye Zhang, Yang Zhang, Wenyan Zhong, Jinjia Zhao, Dehong Zheng, Lifang Ruan

**Affiliations:** 1 National Key Laboratory of Agricultural Microbiology, College of Life Science and Technology, Huazhong Agricultural University, Wuhan, China; 2 State Key Laboratory for Conservation and Utilization of Subtropical Agro-bioresources, Guangxi Key Laboratory of Agro-environment and Agro-product Safety, College of Agriculture, Guangxi University, Nanning, China; The University of British Columbia, Vancouver, British Columbia, Canada; University of Toronto, Toronto, Ontario, Canada

**Keywords:** *Ralstonia solanacearum*, type III effectors, ETI supression, BPA1-ACD11 complex

## Abstract

Effectors secreted by phytopathogenic bacteria can suppress ETI responses induced by avirulence effectors, thereby overcoming crop resistance. However, the detailed mechanisms remain largely unknown. We report that the effector RipD from *Ralstonia solanacearum* regulates plant cell death in a protein abundance-dependent manner. RipD targets *Arabidopsis* BPA1, which directly interacts with the key cell death negative regulator ACD11. RipD competes with ACD11 for binding to BPA1, leading to the selective degradation of BPA1 via autophagy, sparing ACD11. A lower dose of RipD promotes BPA1 degradation but leads to ACD11 accumulation, thereby inhibiting RipAA-induced cell death. Conversely, higher levels of RipD degrade both BPA1 and ACD11, resulting in autophagy-dependent cell death. Visualization of RipD delivery by *R. solanacearum* indicated that it reaches levels sufficient to promote ACD11 accumulation and inhibit cell death. Our study reveals a novel mechanism by which an effector inhibits ETI and, for the first time, highlights the critical role of protein abundance in its function.

**IMPORTANCE:**

*R. solanacearum* infects major economic crops, notably tomato, potato, and tobacco, leading to substantial yield reductions and economic losses. This pathogen utilizes various type III effectors to suppress host resistance, often resulting in weakened or lost resistance. However, the underlying mechanisms remain largely unknown. Here, we reveal a novel mechanism by which RipD targets the BPA1-ACD11 complex, which is involved in host immunity and cell death. RipD regulates ACD11 protein homeostasis in a dose-dependent manner by competitively binding and activating autophagy, thereby modulating plant cell death. Importantly, visualization analysis revealed that the amount of RipD secreted by *R. solanacearum* into host cells is sufficient to inhibit Avr effector-induced cell death. Our study highlights for the first time the critical role of effector dosage, deepening the understanding of how *R. solanacearum* suppresses host ETI-related cell death and providing guidance and resources for breeding bacterial wilt resistance.

## INTRODUCTION

Under the threat of complex and diverse pathogens, plant genomes encode a range of pattern recognition receptors (PRRs) localized on membranes that recognize a series of pathogen-associated molecular patterns (PAMPs) and activate the PAMP-triggered immunity (PTI) to limit bacterial proliferation (1). As a counter defense mechanism, pathogens secrete a repertoire of type III system effectors (T3SEs) into host cells, to suppress the PTI, thereby facilitating proliferation and causing disease (2). Plants employ a process called effector-triggered immunity (ETI) to perceive the presence or activity of some T3SEs (named avirulence genes, Avr genes) by nucleotide-binding and leucine-rich repeat (NLR) receptors, which trigger a robust immune response and confer disease resistance (3). Recently, a series of studies demonstrated that PTI and ETI share similar key immune components and lead to similar downstream responses, forming a complex immune defense system together (4–7).

A robust overcoming of the ETI response by pathogens is one of the prerequisites for successful infection. A pangenome T3SEs analysis revealed that 96.8% of strains of the *Pseudomonas syringae* species complex harbor at least one T3SE or its ortholog, which can induce an ETI response in *Arabidopsis thaliana* Col-0. Moreover, many *P. syringae* strains can effectively infect Col-0, suggesting that they have acquired a robust mechanism to bypass or inhibit the ETI response (8). Pathogens often overcome the crop resistance conferred by NLR genes in a short time (9), indicating that host-adapted pathogens have acquired the ability to evade or suppress ETI.

Phytopathogens can evade ETI by losing, silencing, or functionally impairing the Avr genes. For example, a study of *Leptosphaeria maculans* populations that overcame the resistance conferred by RLM1 in cultivars revealed that 90% of the strains had large deletions in the coding region of the AVR effector AvrLm1 (10). This strategy may only work for functionally redundant effectors. When virulence-essential effectors, such as AvrE, HopM1 and others (11, 12), are recognized by NLR receptors (8), simple loss-of-function mutations of effectors will not provide the pathogen with an invasive advantage by escaping ETI, as these would directly compromise pathogenicity (13). Therefore, ETI-suppression effectors provide an additional strategy for pathogens to suppress the ETI induced by virulence-essential effectors (13), such as the *A. thaliana* NLR protein RPS2 (resistance to *P. syringae* 2), which senses the cleavage of the RPM1-interacting protein 4 (RIN4) by *P. syringae* AvrRpt2 to trigger the ETI response (14). This ETI activation was inhibited by HopF1, which blocked the degradation of RIN4 (15). Additionally, the effector RipAC from *Ralstonia solanacearum* targets SGT1 (suppressor of the G2 allele of skp1) to inhibit ETI. Other effectors, such as RipI, RipAP, and RipAU, can also inhibit the cell death induced by RipAA in *Nicotiana benthamiana* (16). RipV2, another effector from *R. solanacearum*, promotes the degradation of key components in the ETI cascade, EDS1-SAG101-NRG1, through its ubiquitin ligase activity, thereby suppressing the ETI response (17). ETI suppression is a crucial strategy used by pathogens to overcome host resistance. This evasion tactic is observed in multiple effectors across various pathogens (13, 18, 19). However, these mechanisms remain largely unknown.

*Accelerated cell death 11* (*acd11*) in *A. thaliana* encodes a ceramide-1-phosphate (C1P) transfer protein (20), whose mutation leads to autoimmunity and cell death (21). However, complementation of the *acd11* mutant with a C1P transport-deficient variant still partially suppresses cell death (22). The protein stability of ACD11 is maintained by BPA1 (binding partner of ACD11) and its homologous proteins, BPA1-like proteins (BPLs), through direct interaction (23, 24). The *Phytophthora capsici* effector RxLR207 promotes the degradation of BPA1 and BPLs, disrupting the stability of ACD11 and thereby inducing immune activation and cell death (24). *Phytophthora capsici* secretes Rxl207, which degrades ACD11, induces host cell death, and facilitates the transition from the biotrophic phase to the necrotrophic phase, thereby promoting its pathogenicity. However, it remains unknown whether biotrophic pathogens such as *R. solanacearum* can interfere with the BPA1-ACD11 complex to regulate the cell death triggered by recognized effectors.

RipD, a virulence-critical T3SE of *R. solanacearum*, can inhibit immune processes and the hypersensitive response (HR) in *N. benthamiana* (25, 26). However, another report revealed that it has an unstable HR-inducing ability in the same host (27). These opposing phenotypes make it difficult to analyze its function. Here, we determined that the function of RipD in regulating cell death is influenced by its protein abundance. At relatively low levels, RipD can suppress cell death, whereas excessive accumulation of RipD induces cell death. Further mechanistic studies revealed that RipD interacts with the BPA1-ACD11 complex and, through mechanisms involving competitive binding and autophagy activation, differentially regulates ACD11 protein abundance in a RipD dose-dependent manner, thereby modulating plant cell death.

## RESULTS

### RipD is a dual-function effector that induces/inhibits cell death in *N. benthamiana* depending on its protein abundance

**Fig 1:**
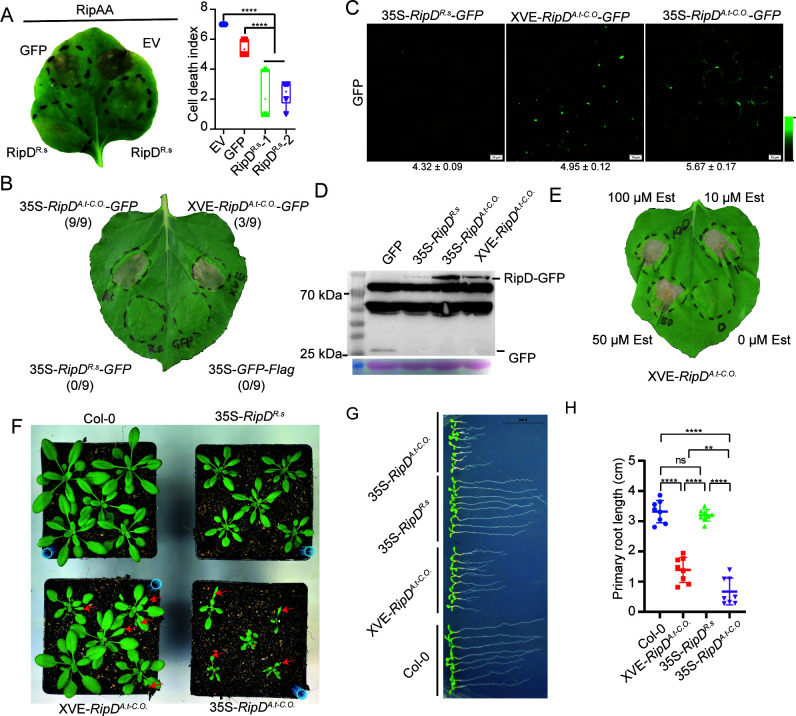
The *R. solanacearum* effector RipD induces cell death and growth inhibition in plants as a function of concentration. (**A**) Cell death inhibition analysis of RipD in *N. benthamiana*. After transient expression of RipD, along with the empty vector and GFP controls, in *N. benthamiana* leaves for 1d, RipAA was subsequently expressed in the corresponding infiltration areas to induce cell death. The phenotype was photographed and scored 4 days after infiltration (dai). The cell death index was calculated with *n* = 6 biological replicates. RipD^R.s^-1 and RipD^R.s^-2 represent two technical replicates at different locations on the same leaf. (**B**) Cell death induction analysis of RipD in *N. benthamiana*. 35S-*RipD^A.t-C.O^-GFP*, 35S-*RipD^R.s^-GFP,* and XVE-*RipD^A.t-C.O^-GFP* were transiently expressed in *N. benthamiana* leaves. Images were captured 5 dai by *Agrobacterium tumefaciens*. (**C**) The protein expression levels were evaluated using confocal fluorescence microscopy. The constructs 35S-*RipD^R.s^-GFP*, XVE-*RipD^A.t-C.O^-GFP*, and 35S-*RipD^A.t-C.O^-GFP* were transiently expressed in *N. benthamiana* and visualized at 2 dai. Means and standard error (SEM) of the GFP intensity are displayed below the image (*n* = 4). (**D**) Western blot assays demonstrated that different levels of RipD were obtained by transient expression of 35S-*RipD^R.s^-GFP*, XVE-*RipD^A.t-C.O^-GFP*, and 35S-*RipD^A.t-C.O^-GFP* in *N. benthamiana*. (**E**) Estrogen enhancesXVE-*RipD^A.t-C.O^* induced cell death. 0, 10, 50, or 100 µM estradiol was applied every 12 h to the inoculated area, followed by *A. tumefaciens* inoculation after 1 day. Images were captured at 5 dai. (**F**) Rosette morphology of 28-day-old wild-type and RipD transgenic *A. thaliana*. The red arrow indicates the deformed leaf. (**G-H**) Lengths of primary roots of 7-days-old wild-type and RipD transgenic *A. thaliana* plants. The quantitative analysis of the primary root lengths is shown in (**H**).

To confirm the function of RipD in activating or inhibiting cell death, we initially transiently expressed *RipD^R.s^* from *R. solanacearum* GMI1000 in *Nicotiana benthamiana. RipD^R.s^* inhibited *RipAA*-induced cell death; however, it did not induce cell death itself (Fig. 1A; Fig. S1A). To increase the expression level of RipD in plants and amplify its phenotype, providing clues for subsequent research, we performed codon optimization on the coding sequence of *RipD^R.s^* for *A. thaliana*, and this gene was designated *RipD^A.t-C.O^*. Unexpectedly, *RipD^A.t-C.O^* elicited robust and stable cell death in *N. benthamiana* (Fig. 1B), which provided us with sufficient grounds to speculate that the protein abundance of RipD is a critical determinant in regulating cell death. To assess the impact of varying protein abundance of RipD on cell death, we further used the XVE promoter to drive the expression of *RipD^A.t-C.O^* (Fig. S1B). The GFP tag was used to visualize the protein abundance of RipD through quantitative fluorescence imaging (Fig. 1C and Fig. S1C) and western blot analysis (Fig. 1D). Unless specifically mentioned, pXVE-*RipD^A.t-C.O^* lines are not induced with estradiol (Est); the accumulation of RipD is occurs through leaky expression (28). The results showed that p35S-*RipD^A.t-C.O^* led to the greatest protein accumulation, followed by pXVE-*RipD^A.t-C.O^*, whereas p35S-*RipD^R.s^* presented the lowest protein abundance. We then transiently expressed these genes in *N. benthamiana* leaves to assay their ability to induce cell death. The results showed that p35S-*RipD^A.t-C.O^* clearly induced cell death, p35S-*RipD^R.s^* did not induce cell death, and pXVE-*RipD^A.t-C.O^* induced unstable cell death (Fig. 1B). Further research reveals that the Est infiltration treatment of pXVE-*RipD^A.t-C.O^* led to a substantial accumulation of RipD, resulting in stable cell death similar to that of p35S-*RipD^A.t-C.O^* (Fig. 1E; Fig. S1D). These results indicate that RipD depends on protein abundance to induce or inhibit *N. benthamiana* cell death.

### RipD inhibited the growth of *A. thaliana* in a dose-dependent manner

All three *RipD* expression constructs were subsequently transformed into *A. thaliana* (Fig. S1E and F). The rosette size, leaf morphology, and root length of *A. thaliana* were used to measure the growth status of the plants. In the T3 generation progeny, p35S-*RipD^R.s^* caused a slight growth delay in the rosette, whereas p35S-*RipD^A.t-C.O^* resulted in more severe growth suppression and partial leaf malformation (Fig. 1F). The expression of RipD at intermediate abundance in pXVE-*RipD^A.t-C.O^* resulted in only minor rosette growth inhibition; however, it caused leaf malformation similar to that observed with p35S-*RipD^A.t-C.O^* (Fig. 1F). The influence of RipD on the primary root growth of *A. thaliana* also depends on protein abundance. p35S-*RipD^R.s^* had negligible effects on the root length of the seedlings, whereas both pXVE-*RipD^A.t-C.O^* and p35S-*RipD^A.t-C.O^* inhibited primary root elongation, with the latter exerting more severe suppression (Fig. 1G and H). We further evaluated whether the substantial accumulation of RipD in Est-induced pXVE-*RipD^A.t-C.O^* lines affects cell death in *A. thaliana*. We found that although 50 µM Est spray treatment induced an increase in RipD accumulation, it did not result in a visible leaf chlorosis phenotype (Fig. S1H). In contrast, infiltration of leaves with 50 µM Est led to greater RipD accumulation and significant leaf chlorosis (Fig. S1H). These results indicate that RipD affects the normal growth and development of *A. thaliana* in a dose-dependent manner, which is in line with the induction of cell death in *N. benthamiana*.

### RipD interacts with BPA1 and its homologous BPL proteins

**Fig 2:**
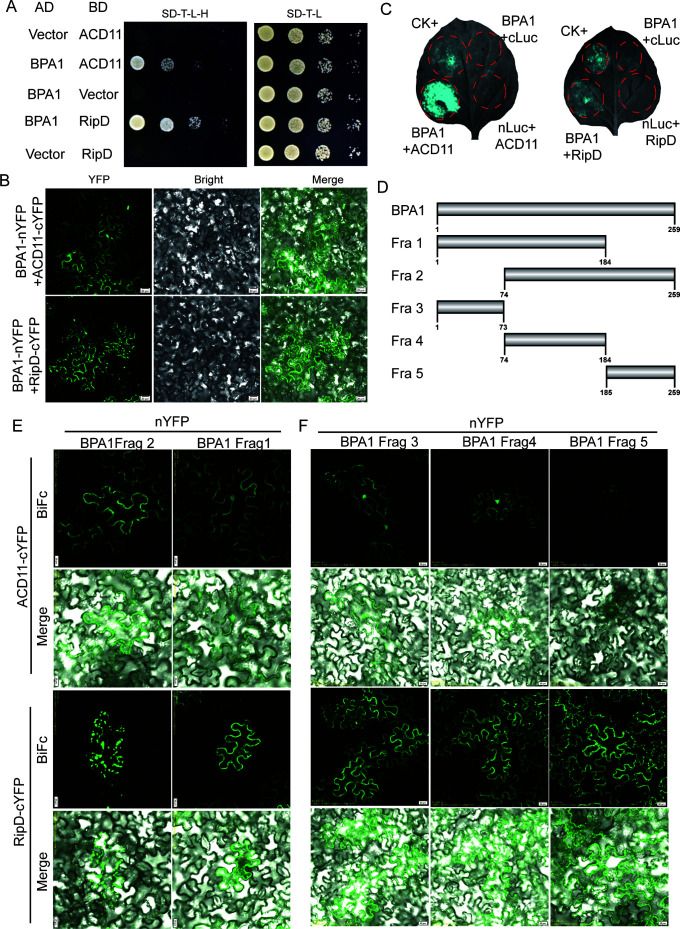
RipD and ACD11 both bind to similar fragments of BPA1. (**A**) Yeast two-hybrid (Y2H) analysis of the interactions between BPA1 and either RipD or ACD11. Clones containing each combination of bait and prey vectors were cultured on both nonselective media (SD/-Trp/-Leu) and selective media (SD/-Leu/-Trp/-His). (**B**) Bimolecular fluorescence (BiFC) assay of the interactions between BPA1 and either RipD or ACD11 in *N. benthamiana* epidermal cells. The YFP signals were observed using confocal microscopy. (**C**) Luciferase complementation imaging assay (LCA) of the interactions between BPA1 and either RipD or ACD11 in *N. benthamiana*. The interaction of Ca1 (AT3G01500) and Ca4 (AT1G70410) was used as a positive control, and n-Luc or c-Luc was used as a negative control. (**D**) Diagram showing the truncation strategy of BPA1. (**E-F**) BiFC analysis of the interactions between various truncated BPA1 fragments and either RipD or ACD11.

To explore the functional mechanism by which RipD regulates cell death in a dose-dependent manner, we first analyzed its target in *A. thaliana*. We performed a yeast two-hybrid (Y2H) library screening and found that RipD interacted with BPA1, which regulated immunity and cell death by interacting with ACD11 (24) (Fig. 2A). The interaction between RipD and BPA1 was further validated by bimolecular fluorescence complementation (BiFC) (Fig. 2B) and luciferase complementation assay (LCA) (Fig. 2C) *in vivo*.

The *A. thaliana* genome encodes six homologs of BPA1, which was previously named BPL1-6 (24). We conducted a Y2H assay using RipD or ACD11 as bait and the BPL proteins as individual prey. Our results showed that BPL1, BPL2, BPL3, and BPL6 all interacted with RipD or ACD11 (Fig. S2A). Unexpectedly, RipD and ACD11 interact with similar BPA1 family members. We further verified the interaction between RipD or ACD11 and BPA1 family members by BiFC. The results showed that BPL1, BPL2, BPL3, BPL4, and BPL5 could interact with RipD or ACD11(Fig. S2B). These results indicate that when homologs of BPA1 interact with ACD11, they are very likely to interact with RipD as well. The consistency of these interactions led us to speculate further that RipD and ACD11 bind to a similar region of BPA1, which determines their interaction with RipD or ACD11.

### RipD and ACD11 bind to an overlapping region of BPA1

To verify the hypothesis that RipD and ACD11 bind to a similar region of BPA1, serial truncation experiments of BPA1 and interaction assays were performed. We designated the truncation of BPA1 based on the protein multiple sequence alignment of BPA1 and BPLs and the protein structure predicted by AlphaFold2 (29) (Fig. S3A and B). The C-terminal region of BPA1, which has high sequence diversity and is predicted to be intrinsically disordered, was removed to obtain fragment 1, and the N-terminal region, which contains a conserved sequence region that is predicted to form an intrinsic structure with a putative RNA-binding function, was removed from BPA1 to obtain fragment 2 (Fig. 2D). BiFc analysis was chosen here because it can simultaneously analyze the ability and subcellular localization of protein-protein interactions. The results revealed that both fragments 1 and 2 could interact with RipD and ACD11 (Fig. 2E; Fig. S3C). We also observed that RipD interacts with different fragments of BPA1 at distinct locations (Fig. 2E). Specifically, RipD predominantly interacts with BPA1 fragment 1 in the cytoplasm and plasma membrane, whereas it mainly interacts with BPA1 fragment 2 in intracellular punctate aggregates. Conversely, the interaction of RipD with full-length BPA1 results in simultaneous localization to the cytoplasm, plasma membrane, and punctate aggregates (Fig. 2B), suggesting a combination of the interaction regions. BPA1 was subsequently divided into fragment 3 at the N-terminus, fragment 5 at the C-terminus, and fragment 4 in the middle, and the ability of these fragments to interact with RipD and ACD11 was analyzed (Fig. 2D). We found that fragments 3 and 4 of BPA1 can interact with RipD and ACD11, but the interaction between BPA1 fragment 5 and ACD11 is greatly reduced compared with that of RipD (Fig. 2F; Fig. S3D and E). These results indicated a significant overlap in the region of BPA1 that mediated the interaction with RipD or ACD11, suggesting a possible competitive binding mechanism.

### Occupancy of the BPA1 binding site by RipD or ACD11 disrupts its interaction with another binding partner

**Fig 3:**
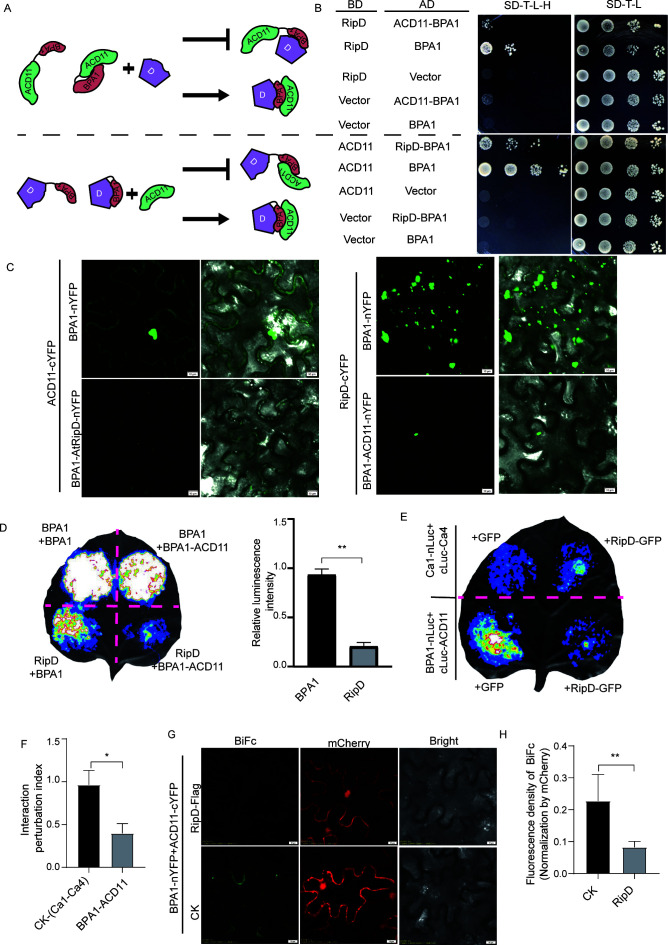
RipD competes with ACD11 for binding to BPA1. (**A**) A schematic illustration of the protein fusion assay was used to assess whether RipD and ACD11 bind to the same region of BPA1. BPA1 and ACD11 were fused by a flexible linker to promote their interaction and increase the occupancy of the interaction surface of BPA1. The interaction between the fusion protein and RipD was subsequently tested. A significantly reduced interaction between RipD and the BPA1-ACD11 fusion protein would indicate that its binding site on BPA1 is occupied by ACD11. Conversely, an unaffected interaction would suggest that the binding site of RipD on BPA1 is distinct from that of ACD11. Additionally, a fusion protein comprising RipD and BPA1 was constructed to examine its interaction with ACD11. (**B**) Comparative analysis of the interactions between RipD and BPA1 or ACD11-BPA was conducted using the Y2H analysis (top). This study also examined the interactions between ACD11 and BPA1or RipD-BPA1(bottom). (**C**) BiFC analysis comparing the interaction strength between ACD11 and BPA1 or RipD-BPA1 (left panel). Similar comparisons were also made for RipD with BPA1 or ACD11-BPA1(right panel). (**D**) LCA analysis comparing the interaction strength between ACD11 and BPA1 or RipD-BPA1. The representative images are shown on the left, and the quantitative analysis results from ImageJ are displayed on the right. The relative luminescence density was calculated as the LUC density of RipD with BPA1-ACD11 divided by its value with BPA1. BPA1 interaction with BPA1-ACD11 or BPA1 was used as a negative control. (**E**) LCA was used to compare the interaction between BPA1 and ACD11 in the presence of GFP-RipD or GFP in *N. benthamiana*. The interaction between Ca1 and Ca4 was used as a negative control. (**F**) The luminescence intensity in (**E**) was quantified by ImageJ, and the ratio of the luminescence density after RipD treatment to that of the GFP control was used as the interaction perturbation index. The data are presented as the mean ± SE (*n* = 3). (**G**) BiFC analysis comparing the interaction between BPA1 and ACD11 co-expressed with RipD-Flag or GUS-Flag (negative control) in *N. benthamiana*. mCherry was utilized as an internal reference. (**H**) Quantification of YFP intensity in (**G**) normalized to the intensity of mCherry. The data are presented as the mean ± SE (*n* = 6).

The truncated BPA1 protein may not reflect the functionality of the complete protein. We designed a protein fusion experiment to further verify that RipD and ACD11 bind to the common region of BPA1. The main scheme is illustrated in Fig. 3A. BPA1 and ACD11 are fused by a Glycine-Serine (GS) linker; owing to their spatial proximity, ACD11 preferentially interacts with BPA1 and occupies the interaction region. If RipD and ACD11 bind to the same region of BPA1, the interaction between the BPA1-ACD11 fusion protein and RipD will be inhibited. Conversely, if RipD and ACD11 bind to different regions of BPA1, the interaction between BPA1-ACD11 and RipD will not be affected (Fig. 3A top). Y2H could be employed to compare the relative strengths of protein-protein interactions when the saturation effect due to the high sensitivity of Y2H is avoided (30). Compared with that of BPA1, the growth rate of the yeast resulting from the interaction between RipD and the BPA1-ACD11 fusion protein was noticeably lower (Fig. 3B top). Similarly, when BPA1 was fused with RipD, the interaction between ACD11 and the BPA1-RipD fusion protein was also reduced (Fig. 3B bottom). These findings preliminarily suggest that RipD and ACD11 may bind to the same region of BPA1. To further substantiate this result, BiFC was employed to assess the interaction strength between the fusion protein and the interactor. BiFC analysis revealed that the YFP signal representing the interaction between ACD11 and the BPA1-RipD fusion protein was reduced compared with that of BPA1 (Fig. 3C left; Fig. S4A). The weakened YFP signal shown in Fig. 3C; Fig. S4A became visible after adjusting the sensitivity of the fluorescence detector to enhance the YFP signal, indicating that the interaction persisted but had become subdued. This finding implied the existence of a reversible competitive binding mechanism (Fig. S4C). As expected, the interaction between RipD and the BPA1-ACD11 fusion protein was also reduced (Fig. 3C right; Fig. S4B). Self-interaction of BPA1 was used as a control to verify that the interaction between the BPA1-ACD11 fusion protein and RipD is inhibited by competitive binding in the common region rather than changes in protein abundance or other factors. The results, as shown in Fig. S4D, indicated that the interaction between BPA1 and the BPA1-ACD11 or BPA1-RipD fusion proteins reached a level similar to that of the self-interaction of BPA1, although the interaction localization between BPA1 and BPA1-RipD was altered. The results of LCA analysis further confirmed the above findings (Fig. 3D): the interaction strength between BPA1 and the BPA1-ACD11 fusion protein was consistent with the self-interaction of BPA1. However, the interaction strength between RipD and BPA1-ACD11 was significantly weaker than that between RipD and BPA1. The above results collectively indicate that the BPA1 binding regions of the effector RipD and the endogenous ACD11 protein of *A. thaliana* overlap, suggesting a potential competitive binding mechanism.

### RipD competes with ACD11 for binding with BPA1 *in vivo*

To further verify whether RipD competes with ACD11 for binding with BPA1, we next analyzed the effects of RipD or GFP on the interaction between BPA1 and ACD11. As expected, we found that RipD significantly inhibited the luciferase activity that represents the interaction strengths between BPA1 and ACD11 in the LCA assay (Fig. 3E and F). However, it did not significantly affect the interaction between Ca1 (carbonic anhydrases 1, *AT3G01500*) and Ca4 (carbonic anhydrases 4, *AT1G70410*), which were used as controls (Fig. 3E and F). Consistent with the above results, RipD, rather than the control, significantly inhibited the YFP signal of the BPA1-ACD11 interaction in the BiFC assay, and mCherry was coexpressed as an internal reference (Fig. 3G and H). In conclusion, our results revealed that the *R. solanacearum* effector RipD competitively binds to the same BPA1 region as ACD11 to form a RipD-BPA1 complex and release ACD11 (Fig. S4E). However, the physiological function of the dissociation of ACD11 from the endogenous BPA1-ACD11 complex is still unclear.

### RipD facilitates plant autophagy activation

Although the quantitative analysis of pathogen-secreted effectors within host cells remains a challenging task, from an energy conservation perspective, the synthesis, unfolding, and secretion of pathogen effectors such as RipD into host cells consume significantly more energy than dose the direct synthesis of BPA1 by the host. Therefore, it is inefficient for RipD to compete directly with the host BPA1 for binding to ACD11. We hypothesize that RipD may also act on the BPA1-ACD11 complex through additional mechanisms.

**Fig 4:**
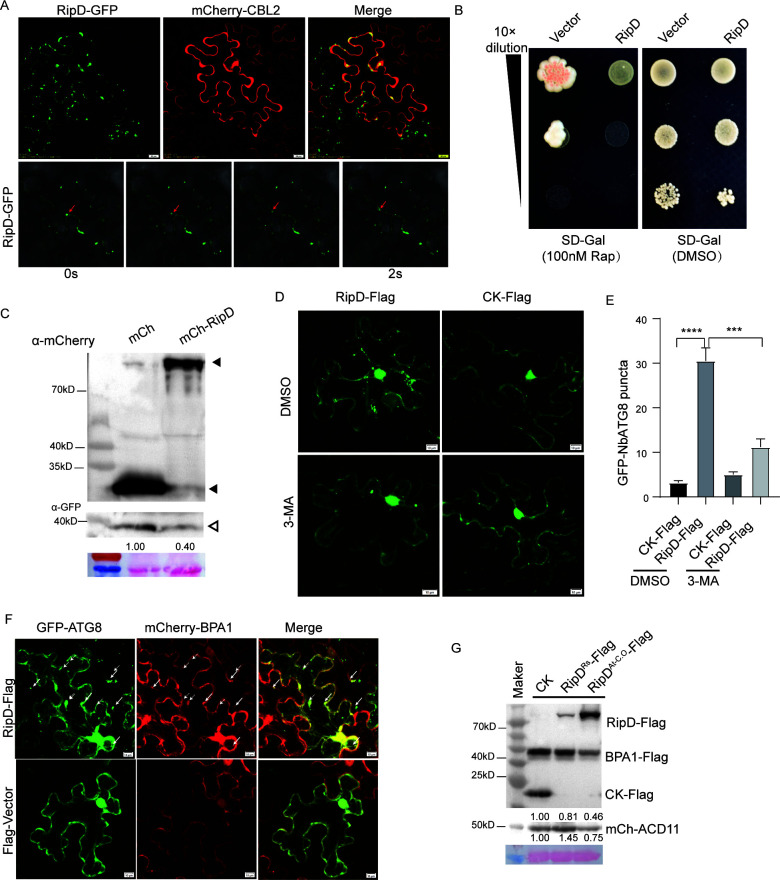
RipD activates autophagy to regulate the protein abundance of BPA1 and ACD11. (**A**) Subcellular localization of RipD-GFP in *N. benthamiana*. Images were taken by confocal microscopy at 2 dai. mCherry-AtCBL2 (*AT5G55990*) served as a marker of vacuoles (top). The location of the RipD-labeled aggregates within 2 s was tracked by time-lapse photography, and the red arrows indicate the moving aggregates (bottom). (**B**) Assay for rapamycin (autophagy inducer) stress tolerance of pGAL-RipD-transformed yeast. Galactose was added to synthetic dextrose (SD) medium (SD-Gal) to induce RipD expression. (**C**) Western blot analysis of the protein abundance of GFP-NbATG8f co-expressing with mCherry-RipD or mCherry in *N. benthamiana* leaves. Ponceau S staining was used as a loading control. The solid triangles on the right indicate the mCherry-RipD or mCherry bands, whereas the hollow triangles point to the GFP-NbATG8f bands. (**D**) Confocal analysis of the effect of RipD-Flag on the accumulation of autophagosomes labeled with GFP-NbATG8f in *N. benthamiana* leaves. 3-Methyladenine (3-MA) was used to inhibit autophagy. (**E**) Quantification of the number of autophagosomes in (**D**). The values represent the means ± SEMs (*n* = 6). (**F**) Autophagosomes induced by RipD colocalized with the mCherry-BPA1 signal, as indicated by the white arrows. (**G**) Western blot analysis showing the effects of different doses of RipD on the abundance of the BPA1 and ACD11 proteins. The numbers below the blots represent the abundance of BPA1-Flag, or mCherry-ACD11 relative to the abundance in the negative control.

Subcellular localization studies revealed that RipD is usually localized in small aggregates when RipD-GFP is transiently expressed in *N. benthamiana* (Fig. 4A), which is consistent with the recently reported phenomenon that *R. solanacearum* infection stimulates the production of small aggregates marked by RipD (25). Notably, some of the labeled RipD aggregates move rapidly within the cell, which may be characteristic of autophagosomes (31). Colocalization of RipD with the vacuole marker CBL2 further enhanced our speculation of its involvement in the autophagy process (32) (Fig. 4A). Interestingly, RipD expression in *Saccharomyces cerevisiae* caused hypersensitivity to the autophagy activator rapamycin (Rap), suggesting that RipD is associated with the autophagy process (Fig. 4B). To further verify the effect of RipD on the plant autophagy process, we coexpressed RipD with GFP-NbATG8f, a popular marker protein for autophagosomes, in *N. benthamiana*. We found that RipD significantly promoted the degradation of GFP-NbATG8f (Fig. 4C) and the formation of autophagosomes (Fig. 4D and E; Fig. S5A), indicating that RipD can promote plant autophagy.

### RipD regulates the protein homeostasis of BPA1 and ACD11

**Fig 5:**
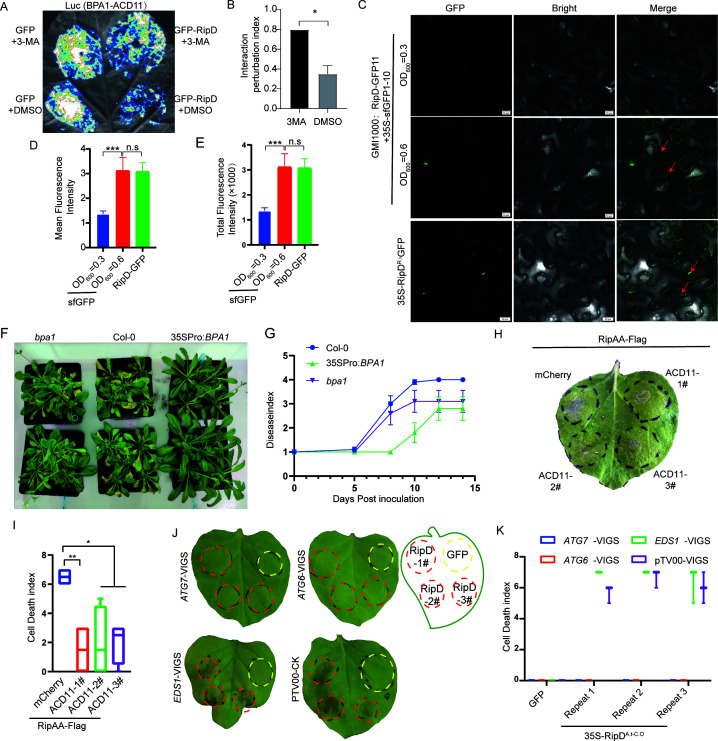
BAP1-ACD11 is involved in plant resistance to *R. solanacearum* and cell death (**A-B**) LCA analysis of the impact of autophagy inhibitors on the ability of RipD to inhibit the formation of the BPA1-ACD11 complex. Leaves were infiltrated with 5 mM 3-MA, with DMSO as a control (**A**) The quantification of the luminescence intensity by ImageJ is shown in (**B**). The data are presented as the means ± SEs (*n* = 3). (**C-E**) Visualization of *R. solanacearum* delivering RipD into plant cells using the sfGFP system. sfGFP1-10 was transiently expressed in *N. benthamiana* leaves, followed by infiltration with *R. solanacearum* expressing RipD-sfGFP11. GFP fluorescence signals were observed by confocal microscopy 3 hours postinoculation. Different densities of *R. solanacearum* were used to analyze the significant accumulation of effectors in host cells during the simulated proliferation of *R. solanacearum*. 35S-RipD^R.s^-GFP was transiently expressed as a control. The mean fluorescence density (**D**) and total fluorescence density (**E**) were quantified using ImageJ. The data are presented as the means ± SEs (*n* = 4). The red arrow indicates vesicular structures labeled with RipD-GFP. (**F-G**) Analysis of *R. solanacearum* resistance in *A. thaliana* overexpressing *bpa1* and in *bpa1* loss-of-function mutants, with wild-type Col-0 used as a control. Representative images at 8 dpi are shown in (**F**), and the results of the disease index analysis throughout the infection process are presented in (**E**). (**H**) Analysis of ACD11-mediated inhibition of cell death in *N. benthamiana*. One day after the transient expression of ACD11-mCherry, RipAA was expressed in the corresponding areas to induce cell death. The cell death phenotype was observed and photographed 2 days postinoculation. (**I**) Quantitative analysis of the cell death phenotype in (**H**) The cell death index was calculated with *n* = 4 biological replicates. Repeats 1–3 represent three technical replicates at different locations on the same leaf. (**J**) Overexpression of RipD induces cell death in an autophagy-dependent manner. GFP or 35S-RipD^A.t-C.O^-Flag was transiently expressed 14 days after VIGS-mediated silencing of the *atg6*, *atg7*, and *eds1* genes in *N. benthamiana,* and the cell death phenotypes were photographed 4 days postinfiltration. Yellow dashe]d circles indicate GFP infiltration areas, whereas red dashed circles mark areas with 35S-RipD^A.t-C.O^-Flag infiltration. (**K**) Quantitative analysis of the cell death phenotype in (**I**) The cell death index was calculated with *n* = 3 biological replicates. Repeats 1–3 represent three technical replicates at different locations on the same leaf.

The mechanism by which RipD employs autophagy to affect the BPA1-ACD11 complex remains unknown. BiFC analysis revealed that the YFP signal, indicative of the interaction between RipD and BPA1, was localized in intracellular punctate aggregates, which was more evident in fragment 2 (Fig. 2B and D; Fig. S5B). These punctate aggregates also moved inside the cells (Fig. S5C), suggesting that RipD might facilitate the degradation of BPA1 by promoting its entry into autophagosomes. To test this hypothesis, RipD, mCherry-BPA1, and GFP-NbATG8f were transiently expressed in *N. benthamiana*. Maximum projections of 3D image stacks were further used to visualize autophagosomes and BPA1. The results revealed that RipD significantly increased the production of intracellular autophagosomes, most of which colocalized with BPA1 (Fig. 4F). Consistent with expectations, RipD promoted the degradation of BPA1, and this degradation was further enhanced with increasing protein abundance of RipD (Fig. 4G). Unexpectedly, the effect of RipD on the abundance of the ACD11 protein differed from that on BPA1; a lower abundance of RipD promoted the accumulation of ACD11, whereas a higher abundance facilitated its degradation (Fig. 4G). To further verify the synergistic effect of RipD-induced autophagy and competitive binding ability, we analyzed the inhibitory effect of RipD on the formation of the BPA1-ACD11 complex undertreatment with the autophagy inhibitor 3-MA. We found that 3-MA significantly inhibited, but did not completely abolish, the ability of RipD to interfere with the BPA1-ACD11 complex (Fig. 5A and B). Our results revealed that RipD can activate autophagy and competitively disrupt the BPA1-ACD11 complex, allowing appropriately abundant RipD (p35S-RipD^R.s^) to facilitate the degradation of BPA1 via autophagosomes while excluding ACD11 from this process. The slight degradation of BPA1 does not lead to destabilization of the ACD11 protein; instead, it promotes the accumulation of ACD11. This intricate dose-regulated mechanism requires further investigation.

### Visualization analysis of RipD delivery by *R. solanacearum* into host cells

Since the influence of RipD on the protein abundance of BPA1-ACD11 is dose-dependent, we first needed to determine the approximate range of RipD protein abundance secreted by *R. solanacearum* into host cells. Quantifying the effector abundance within host cells is a technically challenging task. Fluorescent protein self-assembly systems, such as sfGFP, provide powerful tools for visualizing effectors during infection (33, 34). Briefly, GFP is split into two parts, termed sfGFP1-10 and sfGFP11, neither of which fluoresces individually. SfGFP1-10 is expressed in host cells, whereas sfGFP11 is fused with RipD and expressed in *R. solanacearum* GMI1000. Only after the effector is secreted into the host cell can sfGFP1-10 spontaneously assemble with sfGFP11 to form a complete GFP structure, thereby emitting fluorescence. Here, we used this detection system to quantify the level of RipD secretion during infection. After transient expression of sfGFP1-10 in *N. benthamiana* leaves, different densities of *R. solanacearum* expressing the RipD-sfGFP11 fusion protein infiltrated the corresponding areas. The intensity of GFP fluorescence in the leaves was analyzed 3 hours postinoculation via confocal microscopy, and 35S-RipD^R.s^-GFP transient expression was used as a control. As shown in Fig. 5C, a clear GFP signal was observed on the *N. benthamiana* cell membrane. The GFP signal significantly increased when the inoculation density of the pathogen increased. This may be due to multiple *R. solanacearum* cells injecting effectors into the same host cell, significantly increasing the effector dosage within host cells with increasing pathogen numbers. Notably, when the inoculation density of *R. solanacearum* reached an OD_600_ of 0.6, the sfGFP fluorescence signal reached a level similar to that of transiently expressed 35S-RipD^R.s^-GFP (Fig. 5C through E). This finding indicates that during the infection process, the effector dosage secreted by *R. solanacearum* into host cells can be comparable to that of 35S-RipD^R.s^.

### BPA1-ACD11 is involved in the regulation of *R. solanacearum* resistance and cell death in plants

Moderate-to-low levels of RipD result in slight degradation of BPA1 and the accumulation of ACD11, prompting us to further analyze the impact of BPA1 and ACD11 on cell death and the pathogenicity of *R. solanacearum*. To explore the role of BPA1 in the interaction between *A. thaliana* and *R. solanacearun*, we obtained *A. thaliana* mutants with either loss-of-function or overexpression of *bpa1*. The *BPA1* LOF mutant did not significantly alter *A. thaliana* resistance to *R. solanacearun*, which may be due to functional redundancy between BPLs and BPA1. However, the resistance of the *BPA1-*overexpressing lines to *R. solanacearum* significantly increased (Fig. 5F and G). In addition, *BPA1* overexpressing plants grew better than wild-type plants did after infection (Fig. 5F), which aligns with previous findings suggesting that increased growth vigor contributes to resistance against *R. solanacearum* (35). ACD11 is a negative regulator of immunity and cell death, but the effects of its overexpression are still unclear. The accumulation of ACD11 caused by low levels of RipD may explain its ability to suppress RipAA-induced cell death. To explore this hypothesis, we transiently expressed ACD11-mCherry *in N. benthamiana* and, 24 h later, expressed RipAA, an ETI inducer, in the same area. We found that ACD11 significantly delayed the onset of cell death, whereas BPA1 and the empty vector had no significant effect on cell death (Fig. 5H and I; Fig S5D). The degradation of ACD11 caused by excessive RipD may be associated with RipD-induced cell death. The dependency of RipD-induced cell death on EDS1, a key component of the SA signaling pathway, was investigated given that cell death resulting from ACD11 loss is dependent on it (36). We silenced *eds1* and the key autophagy components *atg6* and *atg7* (37) using VIGS technology and analyzed the ability of excessive RipD to induce cell death. Surprisingly, we found that cell death induced by excessive RipD does not depend on EDS1 but relies on the autophagy components ATG6 and ATG7 (Fig. 5J and K; Fig. S5E and F). These findings indicate that RipD-induced cell death cannot be explained by the destabilization of ACD11 alone but rather depends on its autophagy activation function, which is often associated with cell death (38).

## DISCUSSION

**Fig 6:**
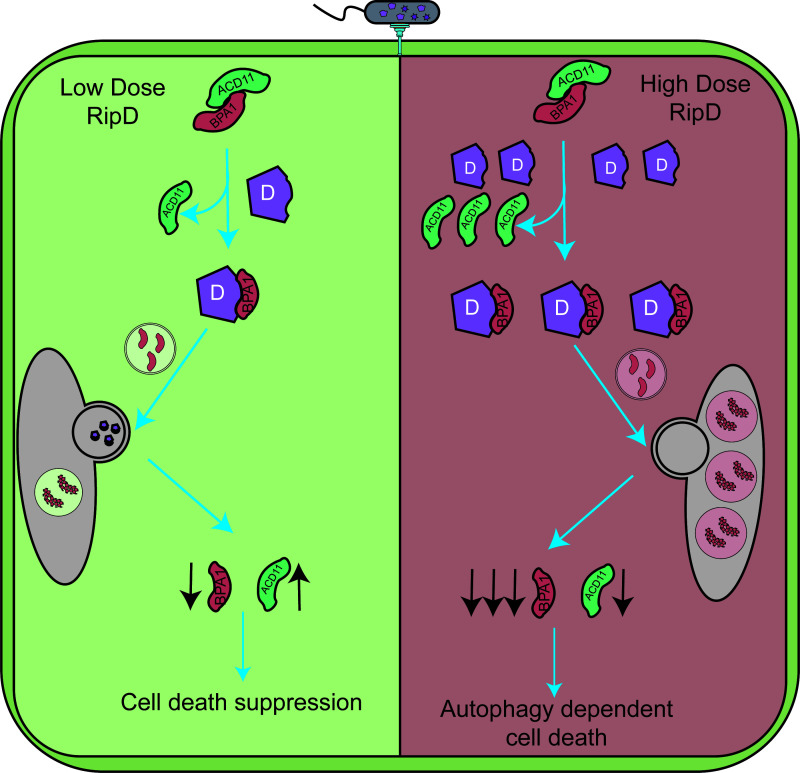
Model of the role of RipD in regulating plant cell death ***R.** solanacearum* secretes relatively low doses of RipD into host cells, where it disrupts the BPA1‒ACD11 complex through competitive binding. RipD promotes the autophagic degradation of BPA1 while excluding ACD11 from this process. This results in moderate degradation of BPA1 and increased accumulation of the ACD11 protein. The accumulated ACD11 inhibits avirulence effector-induced cell death. When excessive RipD enters plant cells, it significantly enhances the degradation of BPA1, thereby disrupting the ability of BPA1 to maintain ACD11 homeostasis, promoting ACD11 degradation, and leading to autophagy-dependent cell death.

Our study identified an effector in *R. solanacearum*, RipD, which inhibits or induces cell death depending on its abundance. RipD, with its competitive binding and autophagy activation functions, promotes the accumulation of ACD11 at appropriate doses to suppress cell death induced by avirulent effectors, thereby facilitating the survival and proliferation of the biotrophic pathogen. This finding reveals a novel mechanism for the suppression of ETI-like cell death. Excessive accumulation of RipD may induce extensive plant autophagy and substantial degradation of the BPA1-ACD11 complex, leading to extensive cell death. Notably, cell death is not entirely due to the significant degradation of ACD11, as it depends on the autophagy process but not on EDS1 (Fig. 6).

### RipD regulates plant immunity and susceptibility to *R. solanacearum* in a dose-dependent manner

Our study reveals the phenomenon and underlying mechanisms by which RipD-mediated regulation of plant cell death is dependent on its abundance. However, we are still unable to determine the effects of RipD abundance on plant immunity and susceptibility to *R. solanacearum*. Other research revealed that a low abundance of RipD, which corresponds to the dose that inhibits plant cell death, stimulates a ROS burst, callose deposition, and the transcription of immunity-related genes in *A. thaliana* (Fig. S6A and E). As the abundance of RipD increases, these immune activation phenotypes are gradually suppressed. Excessive levels of RipD, equivalent to the dose that induces cell death, suppress these immune-related phenotypes (Fig. S6). Specifically, RipD markedly inhibited flg22-induced callose deposition (in pXVE-*RipD^A.t-C.O^* and p35S-*RipD^A.t-C.O^*). Although the constitutive expression of small amounts of RipD (p35S-*RipD^R.s^*) in *A. thaliana* results in callose deposition, a further increase in callose deposition upon flg22 treatment is notably inhibited. This may be related to previous reports that RipD can target vesicle-associated membrane proteins, thereby inhibiting various enzymes and substrates necessary for callose deposition (25). This immune activation phenotype of RipD was unexpected, suggesting that *R. solanacearum* must secrete effectors into host cells at relatively high levels to achieve a balance between immune activation and the suppression of cell death without exceeding the threshold to induce cell death. We subsequently induced RipD expression in the pXVE-*RipD^A.t-C.O^* lines using estradiol spray treatment and found that moderate induction of RipD did not cause obvious cell death (Fig. S1H) but significantly promoted *A. thaliana* susceptibility to *R. solanacearum* (Fig. S6E). These findings indicate that the abundance of RipD secreted by *R. solanacearum* into host cells must reach a certain threshold to balance cell death and immune activation, thereby maximizing virulence. Although we have assessed the effects of various artificial doses of RipD expressed in *A. thaliana* on immunity and resistance to *R. solanacearum*, we are currently unable to determine whether the amount of RipD delivered into host cells during natural infection falls within the range of these experimentally tested doses. However, we speculate that the abundance of RipD during natural infection is currently underestimated owing to the limitations of our effector delivery visualization system, such as the suboptimal assembly efficiency of sfGFP and the inability to observe RipD abundance in host cells after long-term colonization. As RipD is a conserved effector in the *R. solanacearum* species complex and is under strong selective pressure (39), it is plausible that enough RipD is secreted into host cells to balance its functions of immune activation and the suppression of cell death.

### Multilayered suppression of ETI responses by pathogenic bacteria to overcome host resistance

Deploying NLR proteins in crops to recognize pathogen-secreted effectors is currently the most prominent disease resistance breeding strategy (40). However, the resistance conferred by NLRs is often overcome by pathogens within 3–5 years, which is one of the most significant challenges in the breeding of disease-resistant crops (40, 41). Recent studies have shown that pathogens commonly use interactions between effectors, where one effector suppresses the ETI response induced by another avirulent effector, to facilitate infection and proliferation (42).

Effector suppression of ETI primarily operates through two mechanisms: inhibition of AVR effector recognition and suppression of the ETI signaling cascade (42). The inhibition of AVR recognition is often specific; for example, the *P. syringae* effector AvrRPM1 phosphorylates RIN4, which is recognized by RPM1, triggering ETI (43, 44). Another effector, AvrRpt2, cleaves RIN4 and suppresses AvrRPM1-induced ETI (45). The *A. thaliana* NLR protein RPS2 can further recognize AvrRpt2 and activate ETI (46), whereas *P. syringae* employs HopF2 to inhibit AvrRpt2-induced ETI (15). Thus, the recognition and inhibition of AVR effectors by pathogens and hosts follow a coevolutionary ZigZag model (47). Once AVR effectors are recognized by NLRs, the signals converge onto a conserved common signaling cascade (48). Effectors targeting key components within these signaling cascades often broadly suppress ETI responses. For example, the *R. solanacearum* effector RipV2 targets the EDS1-SAG101-NRG1 complex, promoting its degradation and thus broadly suppressing ETI (17). Similarly, RipAC targets SGT1, interfering with the stability of various NLR proteins and broadly suppressing ETI (16). The *P. syringae* effector AvrPtoB interacts with ADR1 and its homologs, promoting their degradation to broadly suppress ETI responses (49).

We report that the *R. solanacearum* effector RipD functions through a novel mechanism to broadly suppress ETI responses and is capable of inhibiting cell death induced by at least two Avr effectors (RipAA and RipE1 (25)). RipD has both inhibitory and death-inducing properties, similar to the previously reported AvrPtoB. AvrPtoB suppresses ETI by degrading ADR1, which is recognized by the *A. thaliana* NLR protein SNC1, thereby activating ETI (49). This likely represents a plant mechanism for protecting key components of its immune system. RipD-induced cell death is not dependent on the classical ETI signaling cascade but relies on autophagy, indicating that plants may mediate protection of the BPA1-ACD11 complex through a different pathway, or this might simply be a form of cytotoxicity.

### ACD11, an important regulator of cell death, is subject to intricate and precise regulation

ACD11 is a ceramide-1-phosphate (C1P) transfer protein that mediates the intermembrane transport of C1P (21). The loss of ACD11 leads to metabolic disruptions in various lipid signaling molecules, such as a dramatic increase in the relative levels of C1P and the pro-cell death molecule phytoceramide, thereby inducing cell death (20). However, the role of ACD11 in regulating cell death cannot be fully explained by its C1P transfer activity, as ACD11 variants lacking C1P transfer activity, as well as the human glycolipid transfer protein GLTP, can partially complement the cell death phenotype caused by ACD11 loss (22). Screening for secondary mutations that suppress cell death induced by ACD11 loss also partially elucidates the mechanism of cell death induction. The absence of ACD11 may increase the transcription of various NLR genes, such as RPS2, RPS4, RPM1, and LAZ5, via the histone methyltransferase LAZ2, thereby inducing immune activation and cell death (50). This finding aligns with the dependency of ACD11-induced cell death on the SA signaling pathway and key ETI components, such as EDS1 and PAD4 (36). Therefore, cell death induced by ACD11 loss is caused by multiple factors, including the regulation of lipid signaling molecules and ETI immune processes. The protein homeostasis and subcellular localization of ACD11 are subject to complex regulation by various factors. The RING-type E3 ligase XBAT35.2 can directly interact with ACD11 and promote its degradation via the 26S proteasome, thereby influencing cell death (51). BPA1 and its homologs, which contain a conserved RNA-binding domain, also interact with ACD11 and help maintain its protein homeostasis (24). In the absence of BPA1 or its homologs, ACD11 undergoes 26S proteasome-dependent degradation. Since BPA1 itself lacks E3 ubiquitin ligase activity, the regulation of ACD11 stability by BPA1 might involve a third protein, possibly XBAT35.2, although the precise mechanism remains unclear. PRA7 and PRA8, reported to interact with both ACD11 and BPA1(23), belong to the prenylated Rab acceptor family involved in the endoplasmic reticulum and vesicle transport processes. VAP27-1, a protein related to vesicle transport and ER-PM contact (52), has also been reported to interact with ACD11 (23). ACD11 interacts extensively with various proteins with different functions, and understanding how these proteins mutually regulate each other to ultimately affect ACD11 function is a highly complex and important task that requires further investigation.

### Limitations

Appropriate doses of RipD cause slight degradation of BPA1 and accumulation of ACD11. We speculate that RipD binding to BPA1 may also interfere with the binding of ACD11 and other regulatory proteins, such as XATB3.5, PRA7, or PRA8, thereby leading to an increase in ACD11 protein abundance. However, the detailed mechanisms remain unclear. Excess RipD leads to autophagy-dependent cell death, which is detrimental to the pathogenicity and proliferation of the biotrophic pathogen *R. solanacearum*. It is unknown whether the level of RipD secreted by *R. solanacearum* into host cells during natural infection can reach this threshold. For our visualization of RipD delivery, we used an interaction system between the nonhost *N. benthamiana* and the *R. solanacearum* strain GMI1000. Extensive cell death prevented longer detection periods for RipD delivery.

## MATERIALS AND METHODS

### Plants and grown conditions

*A. thaliana* and transgenic lines were grown under long-day conditions (16 h day/8 h night) at 23°C. The seeds were sequentially washed gently for 5 min in a 75% ethanol solution containing 0.5% SDS, 5 min in a 75% ethanol solution, and 3 min in absolute ethanol. After drying in a Vertical Flow Clean Bench, the seeds were sown on 1/2 MS medium and grown for approximately 1 week. Then, the seedlings were transplanted to sterile soil and grown until the appropriate time for subsequent experiments. Four-week-old *A. thaliana* plants were used for most experiments in this study.

The seeds of *N. benthamiana* were directly sown on sterile soil and germinated for about 10 days. The seedlings were then transplanted to sterile soil and grown under 23°C and long-day conditions (16 h day/8 h night). The top three fully expanded leaves of 4-week-old *N. benthamiana* plants were used for *Agrobacterium*-mediated transient expression.

### Bacterial and yeast strain culture conditions

*A. tumefaciens* GV3101 and *Escherichia coli* DH5α were cultured in LB medium with appropriate antibiotics, at 28°C and 37°C, respectively, on a shaking incubator. *Ralstonia solanacearum* GMI1000 was cultured in NB medium at 28°C.

The *Saccharomyces cerevisiae* Y187 and AH109 used for yeast two-hybrid assay were cultured in YPDA medium at 30°C. The strains carrying pGADT7 or pGBKT7 plasmids were cultured in SD medium lacking leucine or tryptophan, respectively. The *S. cerevisiae* W303-1A used for heterologous expression of effectors was cultured in a YPDA medium. The strain carrying pYES2 plasmid was cultured in SD medium lacking uracil at 30°C. Ampicillin (100 ng/mL) was added to prevent bacterial contamination.

### Agrobacterium-mediated transient expression and cell death assays

*A. tumefaciens* GV3101 carrying the relevant expression vectors were cultured overnight in LB medium with appropriate antibiotics. Cells were collected by centrifugation and resuspended in infiltration buffer (10 mM MgCl2, 10 mM morpholinoethanesulphonic (MES), pH 5.6, 200 µM acetosyringone) to OD_600_ = 0.5 (about 2.5 × 10^8^ colony-forming units per mL). After incubating at room temperature for 3 h, the *N. benthamiana* leaves were infiltrated using the above bacterial suspension in 1 mL syringe without a needle. The infiltration areas were marked with a black marker. The plants were kept in the dark overnight in the greenhouse and then grown normally until the appropriate time for subsequent experiments. Cell death assays were photographed at 4–5 days after infiltration. The cell death phenotype was categorized into grades from 0 to 7 based on previous reports (53). All instances of cell death were scored, and the results were statistically analyzed and visualized using GraphPad.

### Gene expression analysis

Four-week-old wild-type or transgenic *Arabidopsis* leaves were hand-infiltrated with 100 nM flg22 peptide and harvested at 0 or 1 h after infiltration, then flash-frozen in liquid nitrogen. RNA was extracted using TransZol Up Plus RNA Kit (TransGen Cat No. ER501) according to the manufacturer’s instructions and quantified using NanoDrop ND-1000 UV-Vis Spectrophotometer. RNA was digested with DNAase and synthesized into cDNA using PrimeScriptTM RT Reagent Kit (Takara, Cat No. RR047A) and then diluted 5-fold as a template. One microliter of diluted cDNA, 0.5 µL of primers from Table S1, 3 μL of deionized water, and 5 µL of ChamQ Universal SYBR qPCR Master Mix (Vazyme, Cat No.Q711) were added to 384-well plate (Applied Biosystems, Cat No. 10005724), and real-time fluorescence quantitative PCR was performed using QuantStudio 5 Real-Time PCR System and data were analyzed using QuantStudio Design & Analysis Software. Each sample had four replicates, and the experiment was repeated at least twice and representative results were shown.

### Plant protein extraction and immunoblotting

Plant tissues were collected from *N. benthamiana* leaves 48 h post-agroinfiltration, and ground in liquid nitrogen using TissueMaster High-Throughput Tissue Homogenizer. The total protein extraction buffer (4.5 M urea, 80 µM SDS, 20 µM Tris-HCl (pH = 6.8), and 20 µM β-mercaptoethanol) was added to the tissue sample in 400 µL per 1 mg and thoroughly mixed. After centrifugation at 12,000 g for 10 min twice, the supernatant was collected and mixed with 5× SDS loading buffer and treated in a boiling water bath for 10 min. The protein sample was separated by 10% SDS-PAGE and subsequently transferred onto a nitrocellulose membrane. Ponceau S solution (Biosharp, Cat No. BL519A) was used to ensure equal protein loading. Subsequently, the membrane was treated with a blocking solution of PBST supplemented with 5% (wt/vol) non-fat dry milk. Following this, the membrane was incubated with primary antibodies: anti-GFP (Proteintech, Cat No. 66002–1-Ig) at a dilution of 3.5:10,000, anti-Flag (Proteintech, Cat No. 6008–4-Ig) at a dilution of 1.5:10,000, or anti-mCherry (Proteintech, Cat No. 26765–1-AP) at a dilution of 3.5:10,000. A horseradish peroxidase (HRP)-conjugated secondary antibody (Proteintech, Cat No. SA00001-1) was subsequently applied at a dilution of 3.5:10,000. The incubation period ranged from 40 to 120 min. For chemiluminescence detection of the protein of interest, the membrane was first incubated with SuperSignal West Pico PLUS Chemiluminescent Substrate (ThermoFisher, Cat No. 34580) and then visualized using a Tanon 4600 Chemiluminescent Imaging System (Bio Tanon, China).

### Disease resistance assay

*R. solanacearum* GMI1000 was grown overnight in liquid NB medium in a shaking incubator (200 rpm, 28°C). Cultures were centrifuged at 8,000 rpm for 15 min, and the pellet was collected, washed with sterile water, and then re-suspended in sterile water to adjust the cell density to 10^8^ cfu/mL. Two pots (each containing 5 *Arabidopsis* plants) were soil-soaked inoculated with the bacterial suspension. Disease symptoms were evaluated every 2 days post-inoculation, according to the proportion of diseased leaves in the total leaf proportion. They were classified into levels: 0 (no symptoms), 1 (0%–25%), 2 (26%–50%), 3 (51%–75%), and (76%–100%).

### Yeast two-hybrid assay

The Arabidopsis yeast two-hybrid cDNA library (Y187) was used to mate with RipD-pGBKT7 (AH109) and screen for interacting proteins. A series of potential interacting proteins were identified, and those with functional annotations related to immune regulation were selected for further Y2H pairwise validation. Using the primers in Table S1, the coding regions of the effectors or candidate target genes were amplified by PCR, and homologous arms were added at both ends. They were then cloned into pGBKT7 or pGADT7 vectors using the ClonExpress II One Step Cloning Kit (Vazyme, Cat No.C112). The effectors were used as baits, fused with the GAL4 binding domain, and transformed into yeast AH109. The candidate targets were used as prey, fused with the GAL4 activation domain, and transformed into yeast Y187. The paired baits and preys were mated for 24 h in 2× YPDA medium and enriched in SD medium lacking leucine and tryptophan. Their interaction abilities were evaluated by their growth on SD selective medium (lacking leucine, tryptophan, histidine, and/or adenine).

### Bimolecular fluorescence complementation and subcellular localization assay

The BiFC analysis was performed by transient expression in *N. benthamiana* leaves. The N- and C-terminal fragments of YFP were respectively fused to the paired interacting proteins. For localization, similar to the above, fusion proteins with a GFP or mCherry tag at the C or N terminus were constructed and transformed into *A. tumefaciens. A. tumefaciens* strains were co-infiltrated at a 1:1 ratio into *N. benthamiana* leaves using a 1 mL needleless syringe for BiFC or co-location assay. Samples were taken 36–48 h after agroinfiltration, and the intensity and distribution of the fluorescent protein were observed by laser confocal microscopy. The leaf tissue was observed by FV1000 confocal microscope (Olympus) using 20× air immersion objective (NA = 0.8). The excitation and emission wavelengths of YFP, GFP, and mCherry were 500/535 nm, 488/525 nm, and 560/645 nm, respectively.

### Luciferase complementation assay

Luciferase complementation assay was performed according to previous reports (39). The proteins of interest that interact with each other were fused with either the N-terminal or C-terminal fragment of luciferase, and the constructs were transformed into *A. tumefaciens* GV3101. After 40 h of agroinfiltration, the *N. benthamiana* leaves were coated with 1 mM D-Luciferin (MedChem Express, CAS No. 115144–35-9) containing 0.1% Triton X-100 using a cotton swab, and then kept in the dark for about 10 min. Luciferase imaging was performed using a plant living imaging system (Lumazone Pylon 2048B, Princeton, USA).

### Visualization of effector delivery during *R. solanacearum* infection

This method was primarily based on the protocol by Park et al. (33), with minor modifications. sfGFP1-10 was cloned into the plant expression vector pCambia2306 and transformed into *A. tumefaciens* GV3101 for transient expression in *N. benthamiana* leaves. Concurrently, 35S-RipD-GFP was transiently expressed as a control. RipD-sfGFP11 fusion protein was constructed into the vector pGMI and electroporated into the *R. solanacearum* GMI1000 (*RipD*-deficient). This strain was cultured to OD_600_ = 0.8 (5 × 10^8^ CFU/mL) and resuspended in 10 mM MgCl2 to OD_600_ = 0.6 and OD_600_ = 0.3. The bacterial suspension was infiltrated into the areas of *N. benthamiana* expressing sfGFP1-10 using a needleless syringe. After 3 h, fluorescence images were captured using a confocal microscope.

### Virus-induced gene silencing (VIGS) in *N. benthamiana*

Gene silencing in *N. benthamiana* was performed following a previously described protocol (54) with appropriate modifications. Primers designed using the website (https://vigs.solgenomics.net/) were employed to amplify DNA fragments targeting *atg6*, *atg7*, and *eds1* from *N. benthamiana* cDNA via PCR. These fragments were then cloned into the pTV00 vector and subsequently transformed into *A. tumefaciens* GV3101. *A. tumefaciens* cultures containing pTV00 and pBINTRA6 were mixed at a 1:1 ratio and infiltrated into the two fully expanded true leaves of N. benthamiana seedlings. Silencing efficiency was assessed 3 weeks later by qRT-PCR, followed by transient expression of 35S-*RipD^A.t-C.O^*-Flag to analyze cell death.

### Quantification and statistical analysis

Data analysis and statistical tests were performed using GraphPad Prism 8, and multiple pairwise comparisons were performed by one-way ANOVA. The asterisks in all figures indicate statistical significance (∗, *P* < 0.05; ∗∗, *P* < 0.01; ∗∗∗, *P* < 0.001; ∗∗∗∗, *P* < 0.0001, ns, not significant). The immunoblot bands of western blot and the luminescence intensity of the luciferase complementation assay were processed and quantified using ImageJ. For fluorescence microscopy images, the fluorescence intensity of YFP, GFP, or mCherry was quantified using OlyVIA (Olympus) and FIJI (http://fiji.sc/). The amino acid sequences of BPA1 and BPLs were aligned using MEGA X (55) and subsequently refined with Jalview (56).

## SUPPLEMENTAL MATERIAL

Supplemental figures mbio.03665-24-s0001.docx Fig. S1 to S5.

Fig. S6 mbio.03665-24-s0002.docx RipD regulates the immune response in a dosage-dependent manner.

Table S1 mbio.03665-24-s0003.docx Primer list used in this study.

ASM does not own the copyrights to Supplemental Material that may be linked to, or accessed through, an article. The authors have granted ASM a non-exclusive, world-wide license to publish the Supplemental Material files. Please contact the corresponding author directly for reuse.
