# Supplementary material for: A Ralstonia solanacearum effector regulates plant cell death by disrupting the homeostasis of the BPA1-ACD11 complex
Source: mBio. 2025 Feb 25;16(4):e03665-24. doi: 10.1128/mbio.03665-24 (PMC11980575; doi:10.1128/mbio.03665-24)
Supplement: Supplemental figures — Fig. S1 to S5. [file mbio.03665-24-s0001.docx]

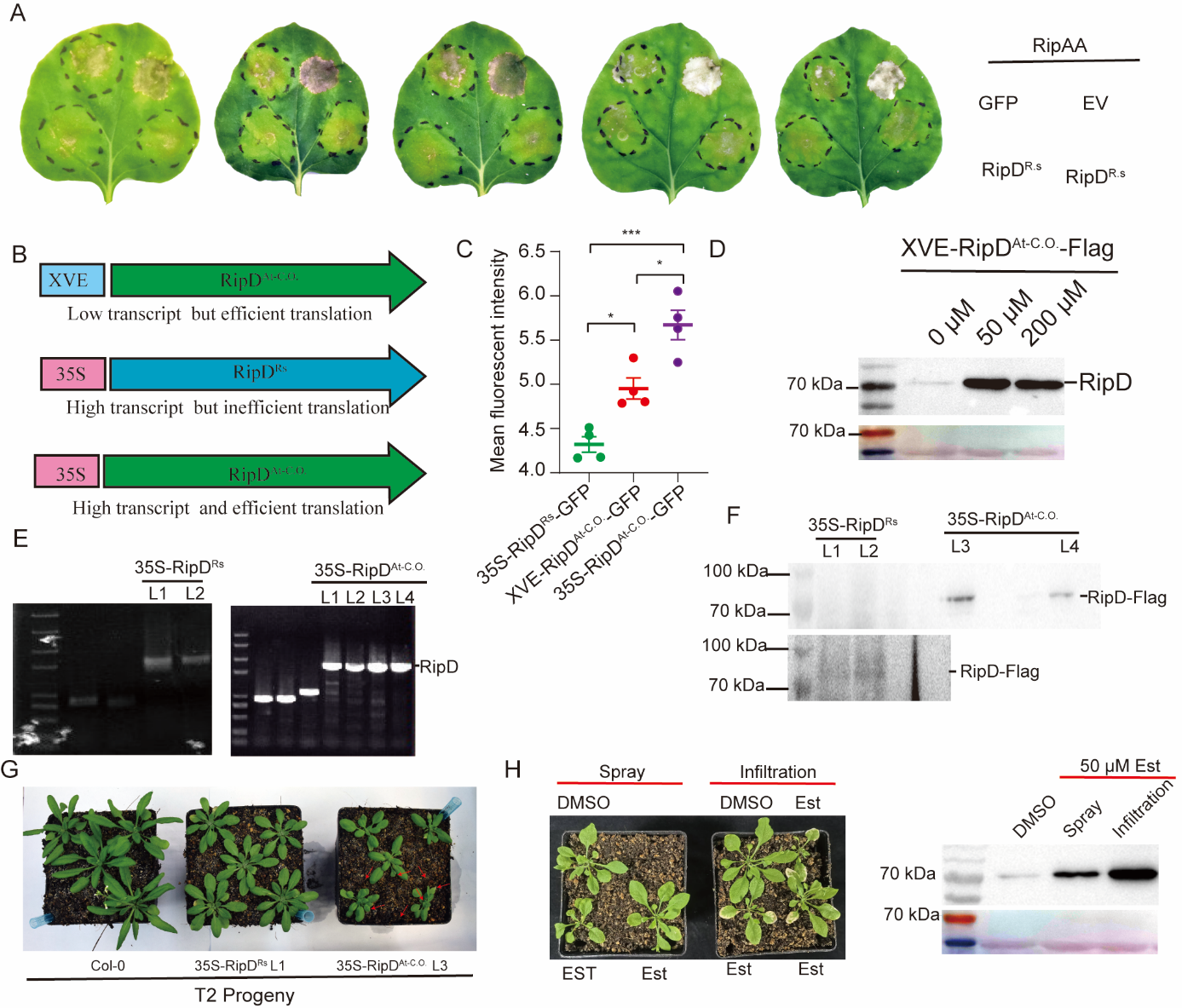


Figure S1, related to Figure 1. Design and validate diverse RipD expression constructs to accumulate varying protein abundances.

(A) A series of biological replicates related to Fig. 1A. Cell-death inhibition analysis of RipD in *Nicotiana benthamiana*.

(B) Schematic diagram of RipD protein abundance controlled by promoter and codon usage bias. The transcriptional activity of the 35S promoter is stronger than that of XVE, and the translation efficiency of the RipD coding sequence optimised for *Arabidopsis thaliana* is higher than that of the unoptimised one in plants.

(C) Quantification of GFP intensity in Figure 1C; the error bars indicate the mean ± SEM for each set (n=4).

(D) Western Blot analysis demonstrated that estradiol treatment significantly increased the accumulation of RipD following transient expression of XVE-*RipD^A.t-C.O^-GFP*.

(E) PCR analysis confirms the transgenic *A. thaliana* with RipD. The gel electrophoresis image shows amplified RipD fragment, confirming its successful insertion into the *A. thaliana* genome.

(F) Western blot analysis confirms the accumulation of RipD protein in transgenic *A. thaliana* lines.

(G) Rosette morphology of 28-d-old wild-type (Col-0) and T2 progeny RipD transgenic *A. thaliana*. The red arrow indicates the deformed leaf.

(H) Est spray or infiltration treatment of *RipD^A.t-C.O^* results in different RipD accumulation and distinct cell death phenotypes. In the left photo, two pots of *A. thaliana* were treated with 50 µM estradiol using spray (left) and infiltration (right) methods, respectively. The phenotypes were observed and photographed 7 days after treatment. The plants in the upper left corner of each pot were treated with DMSO using spray and infiltration methods (marked with yellow circles) as controls. *A. thaliana* showing significant chlorosis after treatment are marked with red arrows. The Western Blot analysis on the right reveals different RipD accumulation due to different Est treatment methods.


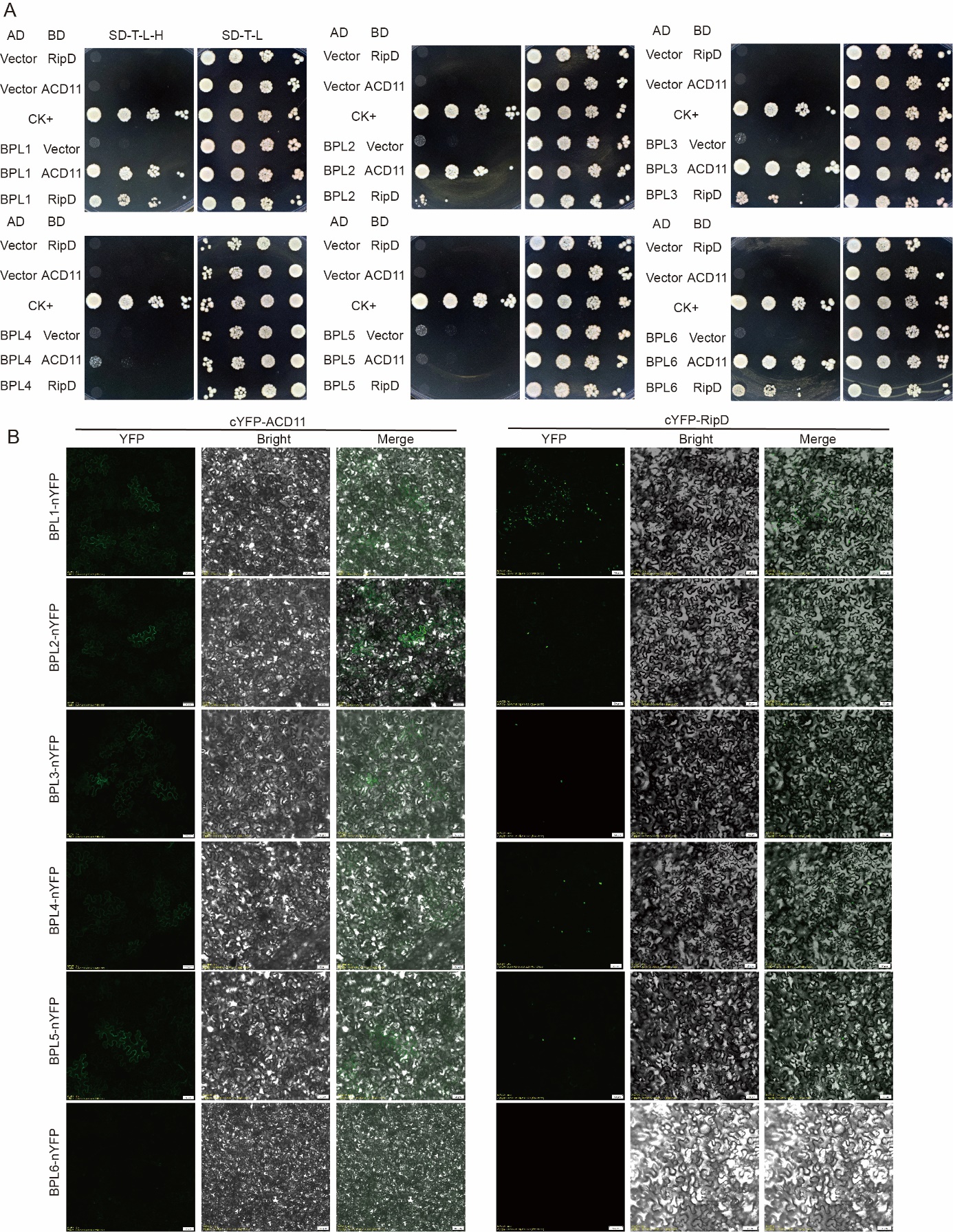


Figure S2, related to Figure 2. RipD interacts with the homologous proteins of BPA1 in a manner analogous to ACD11.

(A) Yeast two-hybrid (Y2H) analysis of the interactions between RipD and ACD11 with BPL1-BPL6. Clones containing each combination of bait and prey vectors were cultured on both nonselective media (SD/-Trp/-Leu) and selective media (SD/-Leu/-Trp/-His).

(B) Bimolecular fluorescence (BiFC) assay of the interactions between RipD and ACD11 with BPL1-6 in *N. benthamiana*.


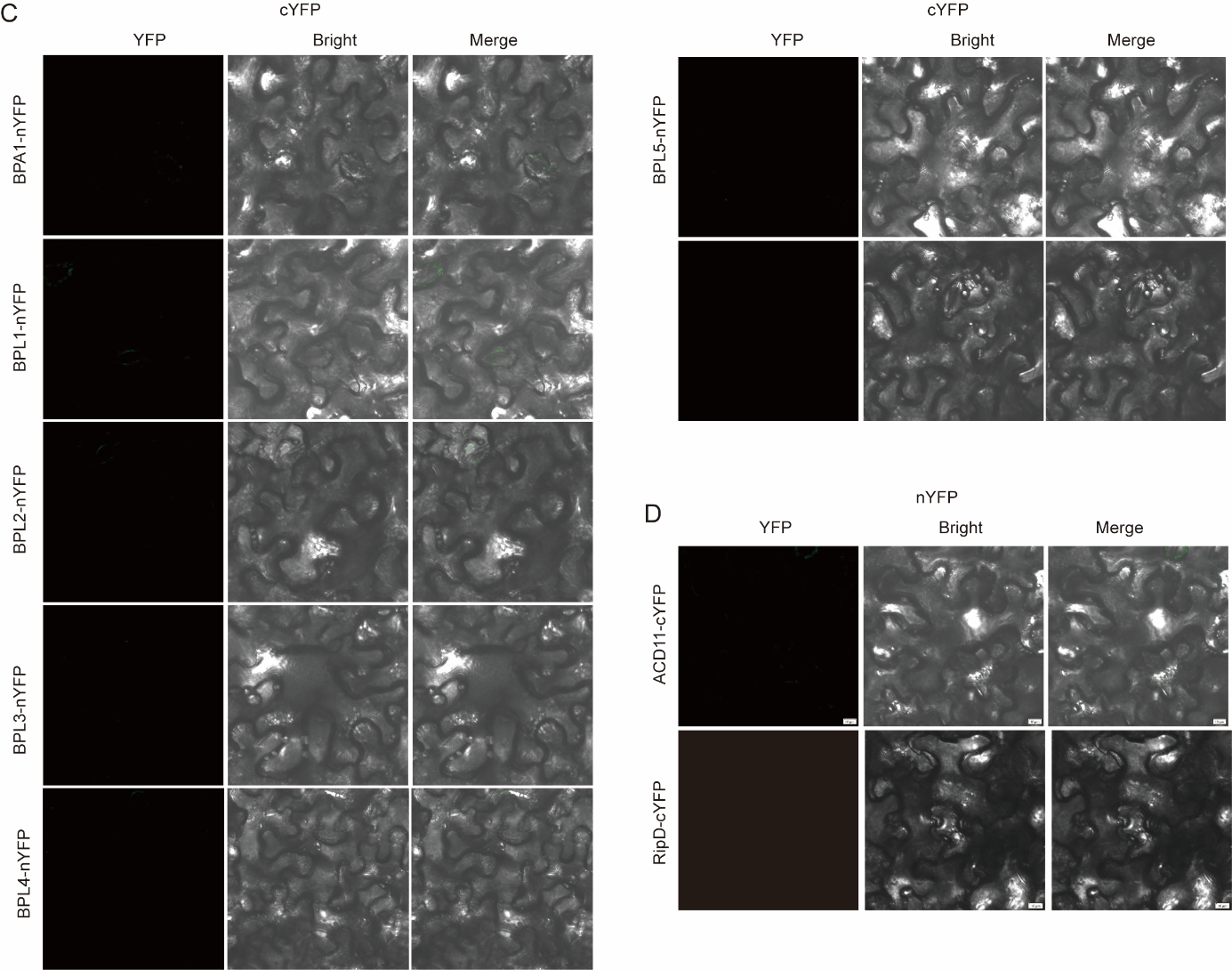


Figure S2-2

(C) Co-expression of cYFP with BPA1 or BPLs did not yield any detectable YFP signal, serving as a negative control for the BiFC analysis.

(D) Co-expression of nYFP with ACD11-cYFP or RipD-cYFP did not yield any detectable YFP signal, serving as a negative control for the BiFC analysis.


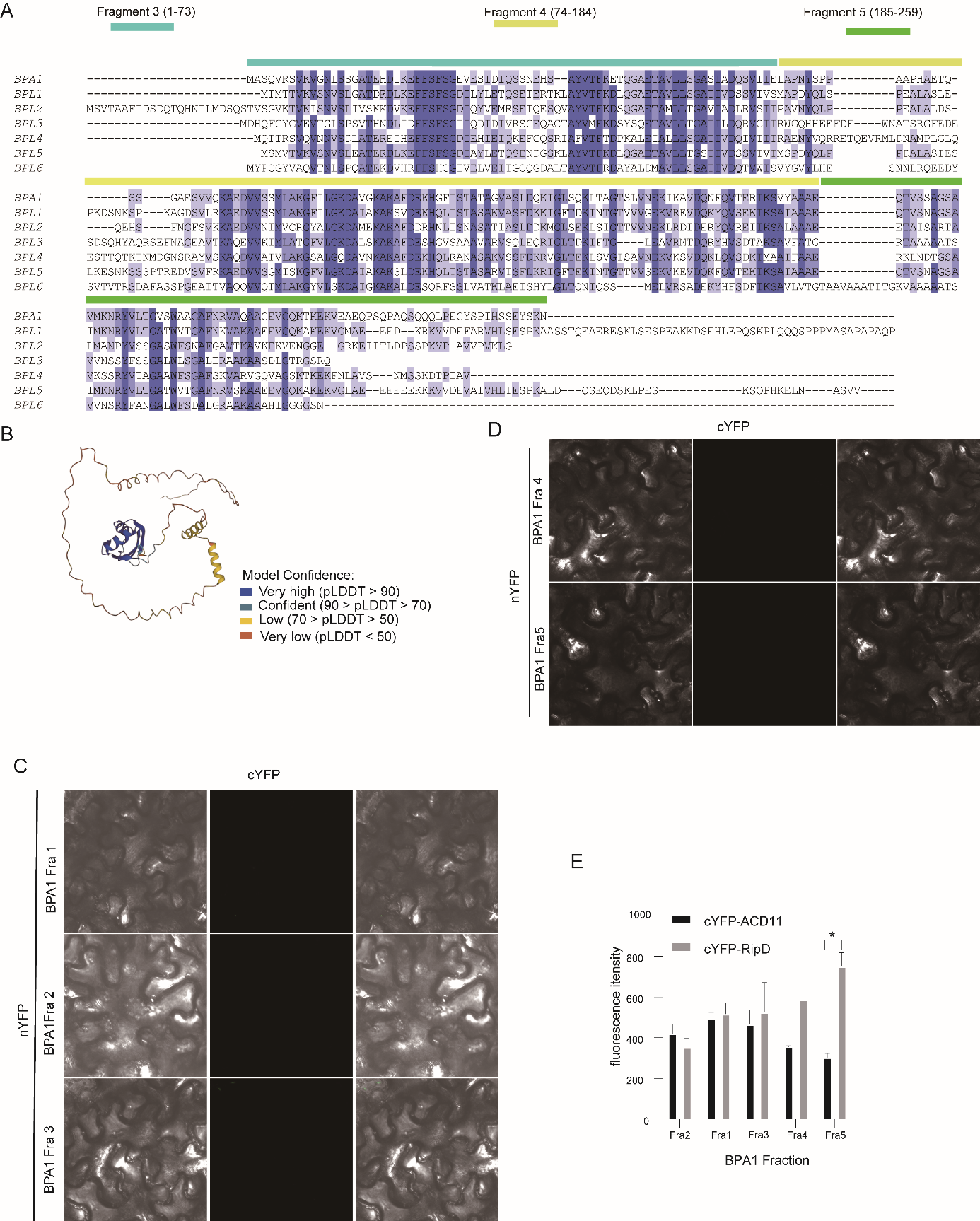


Figure S3, related to Figure 2. The truncation and interaction analysis established the overlapping region of BPA1 that binds to both RipD and ACD11.

(A) Multiple amino acid sequence alignment of BPA1 and its homologous proteins BPL1-BPL6. BPA1 was divided into three segments based on sequence conservation, marked above with blue (fraction 3), yellow (fraction 4), and green (fraction 5), respectively.

(B) The predicted protein structure of BPA1, which was performed by Alphafold2.

(C-D) The co-expression of cYFP with each segment of BPA1 was utilized as a negative control in the BiFC analysis.

(E) In the BiFC analysis, the YFP fluorescence intensity resulting from the interaction of each BPA1 segment with RipD or ACD11 was quantified. the error bars indicate the mean ± SEM for each set (n=6).


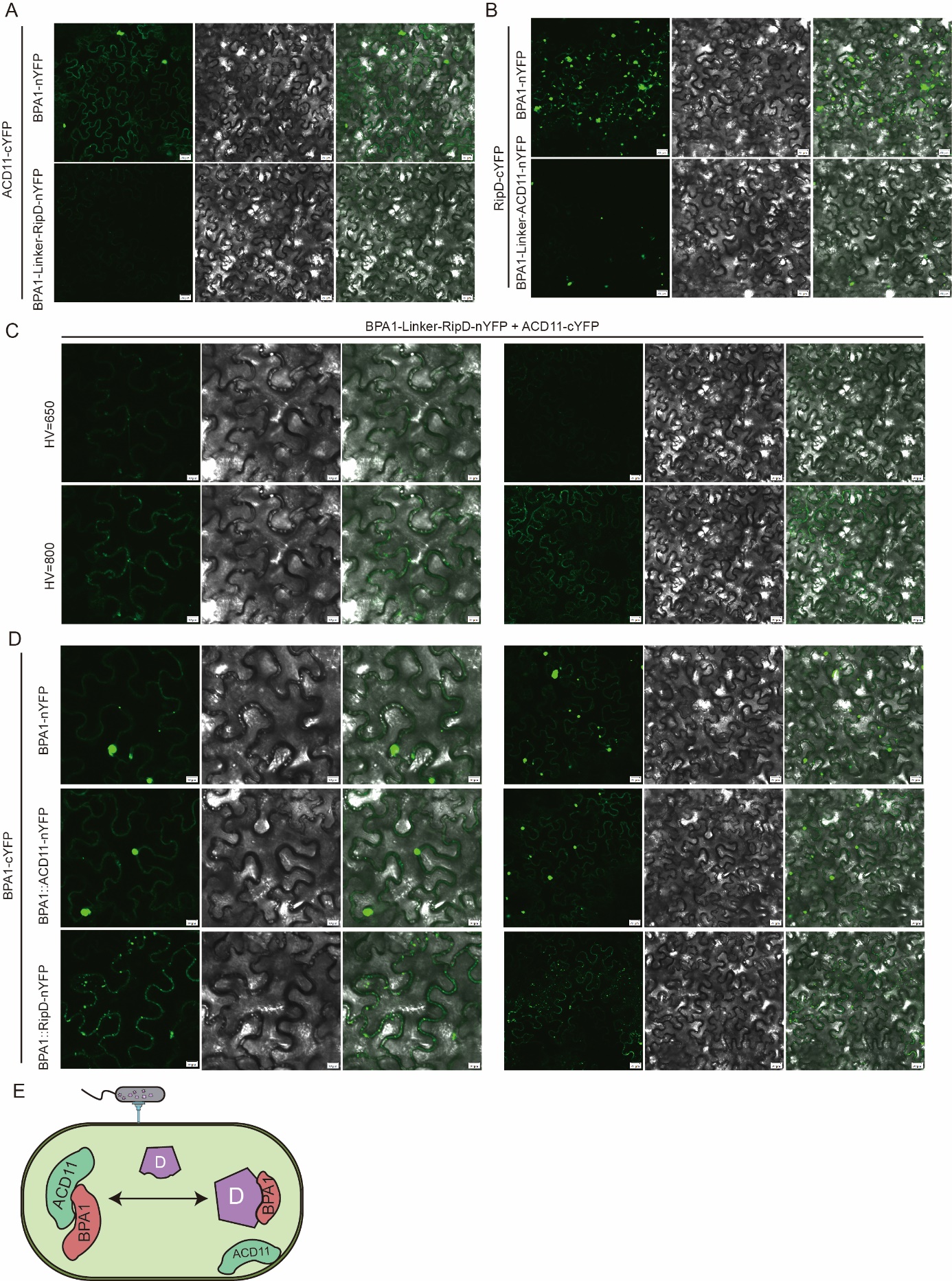


Figure S4, related to Figure 3. The protein fusion analysis demonstrated that RipD and ACD11 bind to the overlapping segments of BPA1.

(A) The interaction strength between ACD11 and BPA1 or BPA1-RipD fusion proteins was compared through BiFC analysis. The central area of the image is magnified fivefold to obtain the left panel of Fig. 3C.

(B) The interaction strength between RipD and BPA1 or BPA1-ACD11 fusion proteins was compared through BiFC analysis. The central area of the image is magnified fivefold to obtain the right panel of Fig. 3C.

(C) The YFP signal representing the interaction between BPA1-RipD fusion protein and ACD11, although weak, was still detectable. ‘HV’ denotes the sensitivity of the confocal microscope’s fluorescence detector; a higher value indicates greater detector sensitivity, resulting in a stronger fluorescence signal.

(D) The interaction between BPA1 and BPA1 fusion protein served as a control to exclude any effects on protein abundance and other factors resulting from the fusion of BPA1 with RipD or ACD11. The interaction between BPA1-ACD11 fusion protein and BPA1 did not exhibit significant changes. Although the localization of the BPA1-RipD fusion protein interaction with BPA1 altered, the YFP fluorescence signal, indicative of the interaction strength, did not show marked changes.

(E) Schematic diagram of RipD competitively inhibiting the interaction between BPA1 and ACD11.


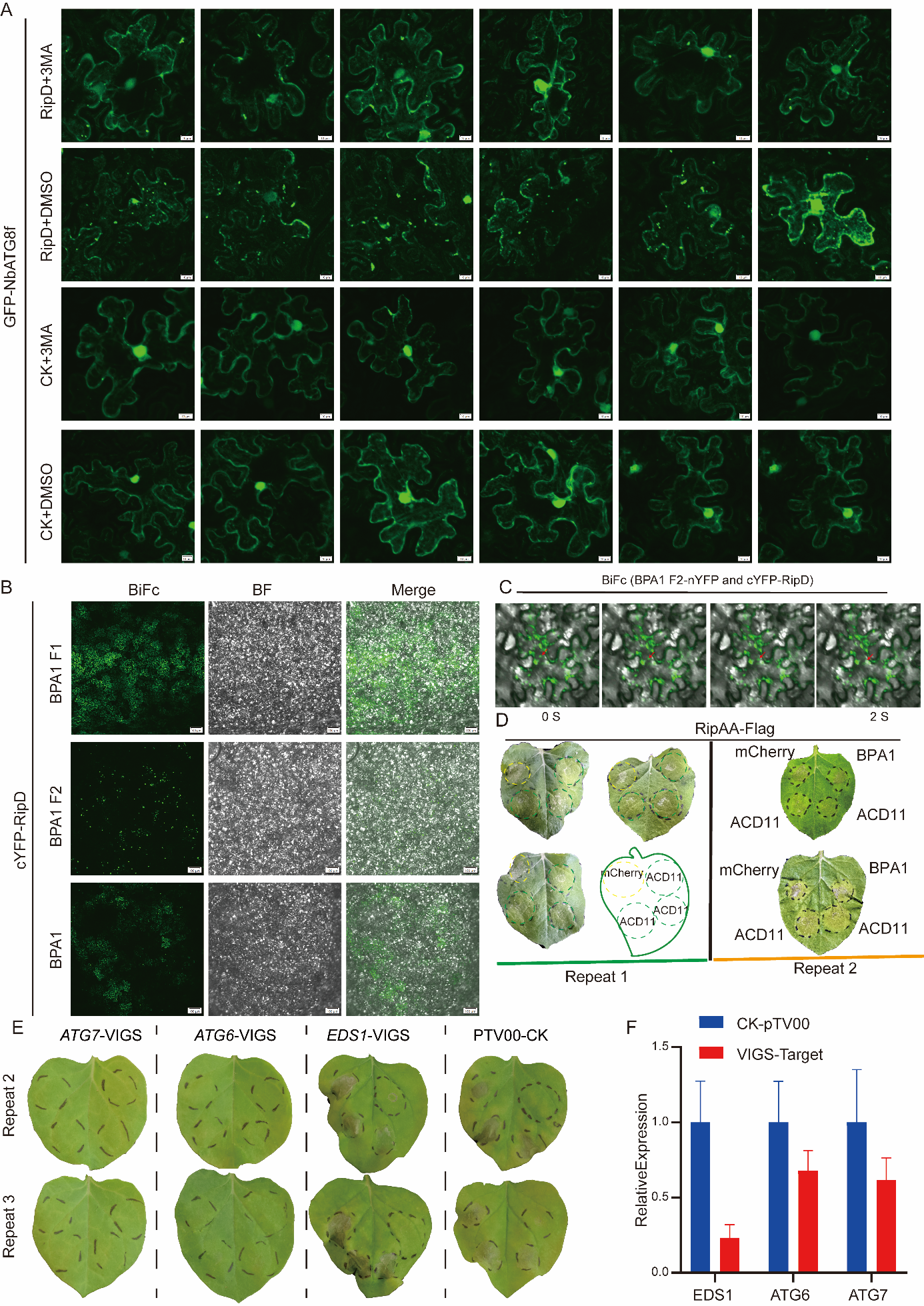


Figure S5, related to figure 4. RipD enhances autophagy and facilitates the entry of BPA1 into autophagosomes.

(A) RipD induces the accumulation of ATG8-labeled autophagosomes in *N. benthamiana*, a phenomenon that is inhibited by the autophagy inhibitor 3-methyladenine (3-MA).

(B) Subcellular localization of the BiFC signal representing the interactions between RipD with BPA1, BPA1-Frac1, or BPA1-Frac2.

(C) The location changes of the BiFC signal-labeled aggregates within 2s were tracked by time-lapse photography, and the red arrows indicate the moving aggregates.

(D) Biological replicates of Fig. 5H show that overexpression of ACD11 significantly inhibits RipAA-induced cell death (left, repeat 1). Analysis of ACD11 and BPA1-mediated inhibition of cell death in *N. benthamiana*. One day after transient expression of mCherry-BPA1 and ACD11-mCherry, RipAA was expressed in the corresponding areas to induce cell death. The cell death phenotype was observed and photographed 2 days postinoculation (repeat 2, right).

(E) Biological replicates of Fig. 5I, analysis of cell death induced by overexpressed RipD after silencing of eds1, atg6, and atg7 in *N. benthamiana*.

(F) The relative expression levels of *eds1*, *atg6*, and *atg7* in VIGS-silenced *N. benthamiana*. The empty pTV00 serves as the control (blue bars)
