# Supplementary material for: A Ralstonia solanacearum effector regulates plant cell death by disrupting the homeostasis of the BPA1-ACD11 complex
Source: mBio. 2025 Feb 25;16(4):e03665-24. doi: 10.1128/mbio.03665-24 (PMC11980575; doi:10.1128/mbio.03665-24)
Supplement: Fig. S6 — RipD regulates the immune response in a dosage-dependent manner. [file mbio.03665-24-s0002.docx]

**RipD regulates the immune response in a dosage-dependent manner**

RipD was observed to inhibit the growth of *A. thaliana*, possibly due to immune activation. Subsequently, we explored the effects of RipD on flg22-triggered Reactive Oxygen Species (ROS) burst, callose deposition, and the expression of immune-related genes. We treated pXVE-*RipD^A.t-C.O^* expression *A. thaliana* with different concentrations of estrogen to control RipD expression level(Figure S6A), and it showed a trend of first enhancing and then reducing flg22-triggered ROS burst (Figure S6A). We therefore speculated whether RipD induces an ETI immune response. Consequently, we monitored the long-term ROS burst co-activated by PTI and ETI. The results showed a similar increase-then-decrease trend in the early ROS burst (0-30 min). Moreover, 50 µM estradiol significantly enhanced the second phase of the ROS burst 2-4 hours after flg22 treatment, suggesting that RipD may possess immune activation functions (Figure S6B). Since the callose deposition is often associated with the burst of ROS, we further quantified the effect of RipD on callose content in *A. thaliana* leaves through aniline blue staining. The findings demonstrate that at 0 hours after flg22 treatment, only the expression of p35S-*RipD^R.s^* led to an increased deposition of callose, whereas the expression of other doses of RipD did not exhibit any notable differences (Figure S6C Top). Compared to Col-0, different doses of RipD exhibited varying degrees of inhibition on callose accumulation 12 h after flg22 treatment, suggesting that one of the primary functions of RipD may be the suppression of callose synthesis (Figure S6C bottom). The various enzymes and substrates required for callose deposition need to be secreted into the apoplast via exocytosis^1^, and RipD interferes with this process through interaction with vesicle-associated membrane proteins, which may be the mechanism by which RipD inhibits callose deposition^2^. The expression of immune-related genes was further analyzed using quantitative real-time PCR. The maker genes for PTI, such as *PR1* and *FRK1*, were significantly induced by p35S-*RipD^R.s^*, regardless of flg22 treatment (Figure S6D). After flg22 treatment, the upregulation of At4g11170 expression in 35S-*RipD^R.s^* was suppressed, which may be related to its involvement in the cell death suppression phenotype.

RipD induced plant immune activation; however, a relatively higher abundance of RipD protein suppressed plant immunity. This study further explored how RipD affects the resistance of *A. thaliana* to *R. solanacearum* GMI1000. The pXVE-*RipD^A.t-C.O^* line was utilized for pathogenicity assays, allowing for the differential expression of RipD controlled by estradiol treatment while ensuring genetic background consistency. We initiated our investigation by inducing protein expression in pXVE-*RipD^A.t-C.O^* *A. thaliana* with estrogen spray treatment, followed by inoculation with GMI1000 and subsequent assessment of the disease index. The findings reveal that the timely expression of RipD, induced by estradiol, significantly heightened the susceptibility of *A. thaliana* to GMI1000 (Figure S6E).


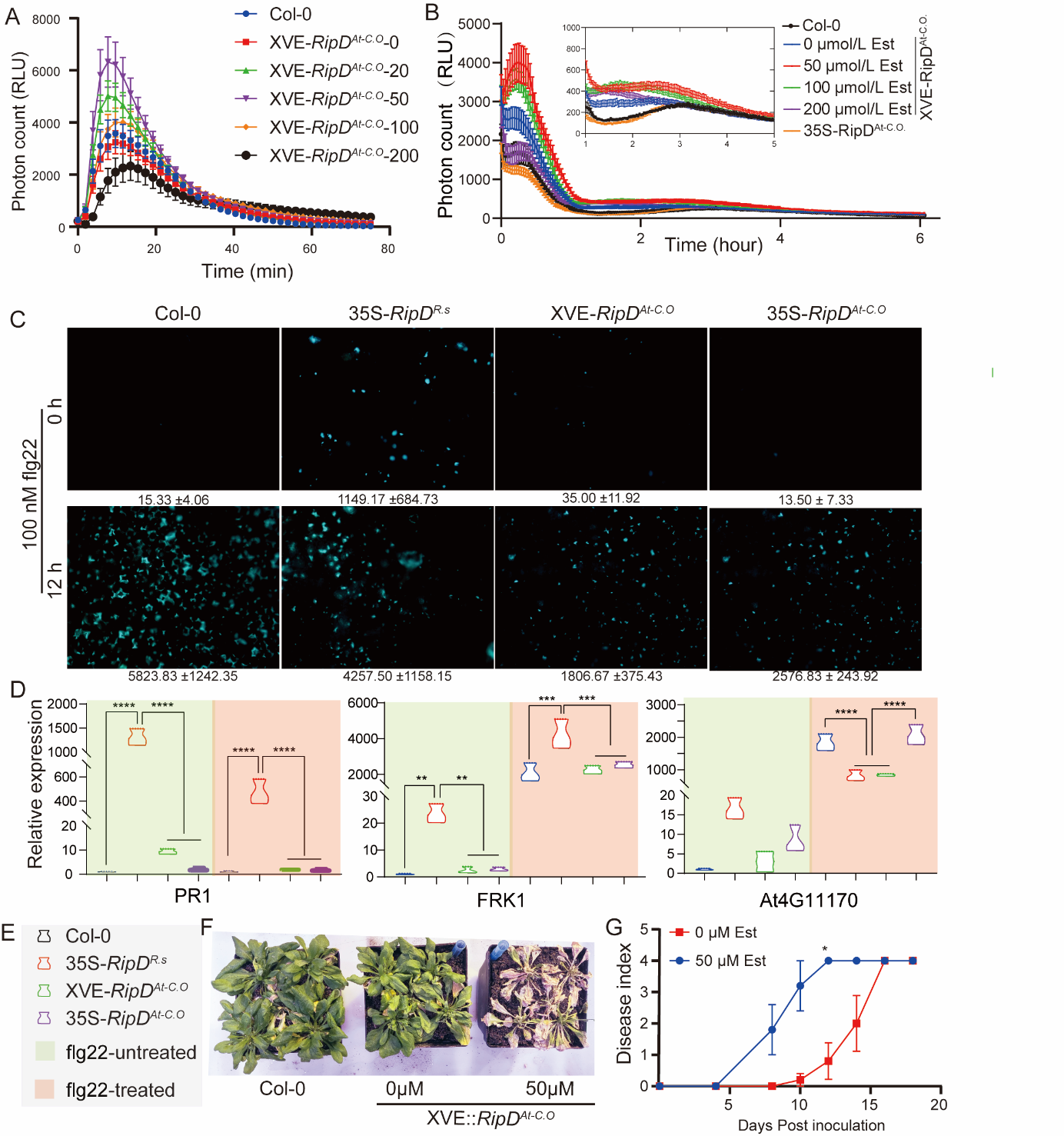


**Figure S6 RipD modulates the immune response in a dose-dependent manner.**

(A) The flg22-induced ROS dynamics in *A. thaliana* leaves expressing the XVE-*RipD^A.t-C.O^* under estrogen treatment. Leaf discs were incubated with 0, 20, 50, 100 or 200 μM of estrogen for 5h before measurement. Data are presented as mean ± SE (n = 16).

(B) Effects of RipD expression and flg22 treatment on the dynamics of long-term ROS burst. Different doses of estradiol were used to treat pXVE-*RipD^A.t-C.O^* to induce RipD expression, with DMSO treatment as a control. Col-0 and 35S-*RipD^A.t-C.O^* represent the absence or overaccumulation of RipD, respectively. Data are presented as mean ± SE (n = 16).

(C) The callose deposition in *A. thaliana* leaves expressing different RipD constructs after 0 or 12 h upon flg22 treatment. Experiments were performed using six leaves harvested from different plants for each genotype. The error bars indicate the mean ± SEM for each set.

(D-E) The quantitative real-time PCR analysis of immune-related gene expression in *A. thaliana* leaves expressing different RipD constructs after 0 or 1 h upon flg22 treatment.

(F-G) The representative image and disease index dynamics of XVE- *RipD^A.t-C.O^* transgenic *A. thaliana* inoculation with *R. solanacearum* GMI1000, which were treated with 0 or 50 μM estradiol for 6 hours before inoculation. The representative picture was taken 14 days post-inoculated.

**METHODS**

**Flg22 triggered ROS assays**

*A. thaliana* leaves were harvested with a cork borer of 5.5 mm in diameter and floated on a 96-well plate containing 200 μL of sterile water in a greenhouse overnight. The next day, the sterile water was aspirated and 100 μL of the reaction solution containing 30 mg/L luminol (MedChem Express, CAS No. 521-31-3), 20 mg/L peroxidase from horseradish (Sigma-Aldrich, CAS No. 9003-99-0) and 1000 nM flg22 peptide (Amino acid sequence: QRLSTGSRINSAKDDAAGLQIA) was added, and the whole process was carried out with care and delicacy to avoid damaging the leaf discs. Immediately after that, the 96-well plate was placed in VICTOR Nivo™ Plate Reader to measure luminescence within 90 minutes or 6 hours.

**Callose staining and quantification**

*A. thaliana* leaves of 4-week-old plants were infiltrated with 100 nM flg22 peptide and harvested at 0 or 12 hours after infiltration. Submerged the leaves in a decolorizing solution (lactic acid: water-saturated phenol: glycerol: ethanol = 1:1:1:6) at 60°C, 200 rpm incubate for 20 min. Then poured out the decolorizing solution, washed the leaves with 50% ethanol twice, and then rinsed with sterile water once. The aniline blue solution (150 mmol/L K2HPO4, pH 9.5, 0.01% aniline blue) was used to dye the cleared leave under dark conditions overnight. The next day, rinsed the leaves with sterile water, observed callose deposits under the Nikon Eclipse 80i Advanced Research Microscope using UV filters. The number of callose deposits was quantified using ImageJ software (<http://imagej.nih.gov/ij>).

Reference

1. Wang, Y., Li, X., Fan, B., Zhu, C., and Chen, Z. (2021). Regulation and Function of Defense-Related Callose Deposition in Plants. Int. J. Mol. Sci. *22*. 10.3390/ijms22052393.

2. Wang, K., Yu, W., Yu, G., Zhang, L., Xian, L., Wei, Y., Perez-Sancho, J., Xue, H., Rufian, J.S., Zhuang, H., et al. (2023). A bacterial type III effector targets plant vesicle-associated membrane proteins. Mol. Plant Pathol. *24*, 1154-1167. 10.1111/mpp.13360.
