# Supplementary material for: A Ralstonia solanacearum effector regulates plant cell death by disrupting the homeostasis of the BPA1-ACD11 complex
Source: mBio. 2025 Feb 25;16(4):e03665-24. doi: 10.1128/mbio.03665-24 (PMC11980575; doi:10.1128/mbio.03665-24)
Supplement: Table S1 — Primer list used in this study. [file mbio.03665-24-s0003.docx]

Table S1：Primer list used in this study

| **Primer Name** | **Primer Sequence 5‘-3’** | **Annotation** |
| --- | --- | --- |
| RipD772-F | TACGCGTCCCGGGGCGGTACCATGGGAAATTTACAGATTAAA | cloning genes for LCA analysis |
| RipD772-R | TGTAGTCCATTTGTTGGATCCGACGCGGTCGTAGTCGATCGA |  |
| BPA1772-F | TACGCGTCCCGGGGCGGTACCATGGCGTCACAGGTAAGGTC |  |
| BPA1772-R | TGTAGTCCATTTGTTGGATCCTTAGTTCTTCGAGTATTCGG |  |
| BPL1771-F | ACGGGGGACGAGCTCGGTACCATGACGATGACAACAGTTAAAG |  |
| BPL1771-R | CGCGTACGAGATCTGGTCGACAGGTTGAGCCGGAGCAGGAG |  |
| BPL2771-F | ACGGGGGACGAGCTCGGTACCATGTCGGTAACGGCTGCTTTC |  |
| BPL2771-R | CGCGTACGAGATCTGGTCGACACCGAGCTTAACAGGAACTAC |  |
| BPL3771-F | ACGGGGGACGAGCTCGGTACCATGGATCATCAATTTGGATATG |  |
| BPL3771-R | CGCGTACGAGATCTGGTCGACCTGCCTTGAGCCGCGGGTAC |  |
| BPL4771-F | ACGGGGGACGAGCTCGGTACCATGCAGACGACGAGAAGTG |  |
| BPL4771-R | CGCGTACGAGATCTGGTCGACTACAGCAATCGGTGTATCC |  |
| BPL5771-F | ACGGGGGACGAGCTCGGTACCATGTCGATGGTAACTGTC |  |
| BPL5771-R | CGCGTACGAGATCTGGTCGACAACAACACTTGCGTTAAGC |  |
| BPL6771-F | ACGGGGGACGAGCTCGGTACCATGTATCCTTGTGGTTACG |  |
| BPL6771-R | CGCGTACGAGATCTGGTCGACATTGCTTCCTCCTCCACCG |  |
| BPA1nYFP-F | GGCGCGCCACTAGTGGATCCATGGCGTCACAGGTAAGGTC | cloning genes for BiFc analysis |
| BPA1nYFP-R | AACTTTTGCTCCATCCCGGGGTTCTTCGAGTATTCGGACG |  |
| ACD11cYFP-F | GGCGCGCCACTAGTGGATCCATGGCGGATTCGGAAGCAGA |  |
| ACD11cYFP-R | TCGTATGGGTACATCCCGGGCCAATCAATACCGAGTTGC |  |
| RipDcYFP-F | GGCGCGCCACTAGTGGATCCATGGGAAACCTTCAGATC |  |
| RipDcYFP-R | TCGTATGGGTACATCCCGGGCACACGGTCGTAATCGATAG |  |
| BPA1cYFP-F | GGCGCGCCACTAGTGGATCCATGGCGTCACAGGTAAGGTC |  |
| BPA1cYFP-R | TCGTATGGGTACATCCCGGGGTTCTTCGAGTATTCGGACG |  |
| BPL1nYFP-F | GGCGCGCCACTAGTGGATCCATGACGATGACAACAGTTAAAG |  |
| BPL1nYFP-R | AACTTTTGCTCCATCCCGGGAGGTTGAGCCGGAGCAGGAG |  |
| BPL2nYFP-F | GGCGCGCCACTAGTGGATCCATGTCGGTAACGGCTGCTTTC |  |
| BPL2nYFP-R | AACTTTTGCTCCATCCCGGGACCGAGCTTAACAGGAACTAC |  |
| BPL3nYFP-F | GGCGCGCCACTAGTGGATCCATGGATCATCAATTTGGATATG |  |
| BPL3nYFP-R | AACTTTTGCTCCATCCCGGGCTGCCTTGAGCCGCGGGTAC |  |
| BPL4nYFP-F | GGCGCGCCACTAGTGGATCCATGCAGACGACGAGAAGTG |  |
| BPL4nYFP-R | AACTTTTGCTCCATCCCGGGTACAGCAATCGGTGTATCC |  |
| BPL5nYFP-F | GGCGCGCCACTAGTGGATCCATGTCGATGGTAACTGTC |  |
| BPL5nYFP-R | AACTTTTGCTCCATCCCGGGAACAACACTTGCGTTAAGC |  |
| BPL6nYFP-F | GGCGCGCCACTAGTGGATCCATGTATCCTTGTGGTTACG |  |
| BPL6nYFP-R | AACTTTTGCTCCATCCCGGGATTGCTTCCTCCTCCACCG |  |
| BPA1nYFP-1 | GGCGCGCCACTAGTGGATCC ATGGCGTCACAGGTAAGGTC | The truncation of BPA1 and its subsequent cloning were conducted for BiFC analysis |
| BPA1nYFP-2 | AACTTTTGCTCCATCCCGGG CTGCTCCGCAGCTGCATAC |  |
| BPA1nYFP-3 | GGCGCGCCACTAGTGGATCC ATGATCATTGAGTTGGCTCCCAAC |  |
| BPA1nYFP-4 | AACTTTTGCTCCATCCCGGGG TTCTTCGAGTATTCGGACG |  |
| BPA1nYFP-5 | AACTTTTGCTCCATCCCGGG CTCATCAAAAGCTTTTGC |  |
| BPA1nYFP-6 | AACTTTTGCTCCATCCCGGG GACTGATTGATCGGCAATAC |  |
| BPA1nYFP-7 | GGCGCGCCACTAGTGGATCC ATGACTGTGAGCAGTGCAGGAAG |  |
| BPL1AD-F | GTACCAGATTACGCTCATATGATGACGATGACAACAGTTAA | cloning genes for Y2H analysis |
| BPL1AD-R | ACGATTCATCTGCAGCTCGAGTCAAGGTTGAGCCGGAGCAG |  |
| BPL2AD-F | GTACCAGATTACGCTCATATGATGTCGGTAACGGCTGCTTT |  |
| BPL2AD-R | ACGATTCATCTGCAGCTCGAGTCAACCGAGCTTAACAGGAA |  |
| BPL3AD-F | GTACCAGATTACGCTCATATGATGGATCATCAATTTGGATA |  |
| BPL3AD-R | ACGATTCATCTGCAGCTCGAGTCACTGCCTTGAGCCGCGGG |  |
| BPL4AD-F | GTACCAGATTACGCTCATATGATGCAGACGACGAGAAGTGT |  |
| BPL4AD-R | ACGATTCATCTGCAGCTCGAGTTATACAGCAATCGGTGTAT |  |
| BPL5AD-F | GTACCAGATTACGCTCATATGATGTCGATGGTAACTGTCAA |  |
| BPL5AD-R | ACGATTCATCTGCAGCTCGAGTCAAACAACACTTGCGTTAA |  |
| BPL6AD-F | GTACCAGATTACGCTCATATGATGTATCCTTGTGGTTACGT |  |
| BPL6AD-R | ACGATTCATCTGCAGCTCGAGTCAATTGCTTCCTCCTCCAC |  |
| ACD11BD-F | TCAGAGGAGGACCTGCATATGatggcggattcggaagcaga |  |
| ACD12BD-R | TCGACGGATCCCCGGGAATTCtcaccaatcaataccgagtt |  |
| BPA1nYFP-1 | GTACCAGATTACGCTCATATG ATGGCGTCACAGGTAAGGTC | Fusion of BPA1 with ACD11 or RipD was performed for Y2H analysis. |
| BPA1nYFP-2 | GGATCCACTTCCAGAACCGGATCCAGAACC GTTCTTCGAGTATTCGGACG |  |
| RipDnYFP-3 | GGTTCTGGATCCGGTTCTGGAAGTGGATCC ATGGGAAATTTACAGATTAAAG |  |
| RipDnYFP-4 | TACGATTCATCTGCAGCTCGAG TCAGACGCGGTCGTAGTCG |  |
| ACD11nYFP-3 | GGTTCTGGATCCGGTTCTGGAAGTGGATCC ATGGCGGATTCGGAAGCAG |  |
| ACD11nYFP-4 | TACGATTCATCTGCAGCTCGAG TCACCAATCAATACCGAG |  |
| pGreenRipD-F | GAGAACACGATCGATAAGCTTATGGGAAATTTACAGATTAA |  |
| pGreenRipD-R | TTTACTCATACTAGTGGATCCGACGCGGTCGTAGTCGATCG |  |
| BPA1mcherry-F | GAGCTCGGTACCCGGGGATCCATGGCGTCACAGGTAAGGTC |  |
| BPA1mcherry-R | CTTGCATGCCTGCAGGTCGACTTAGTTCTTCGAGTATTCGG |  |
| CBL2mcherry-F | GAGCTCGGTACCCGGGGATCCATGTCGCAGTGCGTTGACGG |  |
| CBL2mcherry-R | CTTGCATGCCTGCAGGTCGACTCAGGTATCTTCAACCTGAG |  |
| AtRipDmCherry-F | CTGTACAAGGAGCTCGGTACCATGGGAAACCTTCAGATCAAG |  |
| AtRipDmCherry-R | CTTGCATGCCTGCAGGTCGACTCACACACGGTCGTAATCGA |  |
| AtRipDFlag-F | ACGGGGGACGAGCTCGGTACCATGGGAAACCTTCAGATC |  |
| AtRipDFlag-R | GGCGAATTGGTCGACTCTAGACACACGGTCGTAATCGATAG |  |
| RipDFlag-R | GGCGAATTGGTCGACTCTAGAGACGCGGTCGTAGTCGATCG |  |
| RipDFlag-R | GGTACCCGGGGATCCTCTAGAGACGCGGTCGTAGTCGATCG |  |
| BPA1Flag-F | ACGGGGGACGAGCTCGGTACCATGGCGTCACAGGTAAGGTC |  |
| BPA1Flag-R | GGCGAATTGGTCGACTCTAGAGTTCTTCGAGTATTCGGACG |  |
| At3G04220-NF | CTGATGATACAGGCAGTAGAAGTG | Used for qRT-PCR |
| At3G04220-NR | CTGAAGATTAGACATTCCTCGG |  |
| At2G32140-NF | CGATACACCAGAAGTTTCGACG |  |
| At2G32140-NR | GTCGTCATCTTCATCGTCG |  |
| At4G11170-NF | CTCAAAATTGGGACAATGAAGC |  |
| At4G11170-NR | TCCATCTTTGCAATGTGAGC |  |
| PR1_qPCR-F | ATACACTCTGGTGGGCCTTACG |  |
| PR1_qPCR-R | TACACCTCACTTTGGCACATCC |  |
| FRK1_qPCR-F | CGGTCAGATTTCAACAGTTGTC |  |
| FRK1_qPCR-R | AATAGCAGGTTGGCCTGTAATC |  |
| RbohD_qPCR-F | CGAATGGCATCCTTTCTCAATC |  |
| RbohD_qPCR-R | GTCACCGAGAGTGCGGATATG |  |
| ICS1_qPCR-F | CAATTGGCAGGGAGACTTACG |  |
| ICS1_qPCR-R | GAGCTGATCTGATCCCGACTG |  |
| Actin1-F | CGATGAAGCTCAATCCAAACGA |  |
| Actin1-R | CAGAGTCGAGCACAATACCG |  |
| qAtRipD-F | GCTGCTGAGGGAATCAAAGG | Used for RT-PCR |
| qAtRipD-R | CCAAACACGACGGAGGAAAG |  |
| RipD-sfGFP11-F | agcggtgttggtttcctcgag ggaaagcgggcaggacag | Clone RipD-sfGFP11 into the pGMI vector |
| RipD-sfGFP-R | GCCCGACGACCCGGAGGACCC gacgcggtcgtagtcgatc |  |
| pGMIsfGFP11-F | agcggtgttggtttcctcgag GGGTCCTCCGGGTCGTC |  |
| pGMIsfGFP11-R | caaattccgcggcttatccac TCACGTGATGCCCGCCGC |  |
| sfGFP-F | GAGCTCGGTACCCGGGGATCC ATGTCGAAGGGAGAGGAGC | Clone sfGFP1-10 into the pCambia2306 vector |
| sfGFP-R | GGCGAATTGGTCGACTCTAGA TTATTTTTCGTTCGGATCCTTAGAAAGCAC |  |
| atg6VIGS-F | ACGGTACCTGTCAGAACTGCCACAATC | Clone eds1, atg6 and atg7 fragment into the pTV00 vector |
| atg6VIGS-R | CGCGGATCCTTTATACACTGAAGCAGC |  |
| atg7VIGS-F | ACGGTACCAAGCTCAACAAGTTGCGC |  |
| atg7VIGS-R | CGCGGATCCCTCCTCAACTTTGCCCGAG |  |
| eds1VIGS-F | GGGGTACCGTACAGTTGTAGCACTTCTTTTGGAGAGAAAAAATC |  |
| eds1VIGS-R | CGGGATCCCATCCAAAAGTTATACAGTATGGACAAACCAGG |  |
